# Predicting the Nonlinear Response of PM_2.5_ and Ozone to Precursor Emission Changes with a Response Surface Model

**DOI:** 10.3390/atmos12081044

**Published:** 2021-08-14

**Authors:** James T. Kelly, Carey Jang, Yun Zhu, Shicheng Long, Jia Xing, Shuxiao Wang, Benjamin N. Murphy, Havala O. T. Pye

**Affiliations:** 1 Office of Air Quality Planning and Standards, U.S. Environmental Protection Agency, Research Triangle Park, Durham, NC 27711, USA;; 2 School of Environment and Energy, South China University of Technology, Guangzhou Higher Education Mega Center, Guangzhou 510006, China;; 3 State Key Joint Laboratory of Environmental Simulation and Pollution Control, School of Environment, Tsinghua University, Beijing 100084, China;; 4 State Environmental Protection Key Laboratory of Sources and Control of Air Pollution Complex, Beijing 100084, China; 5 Center for Environmental Measurement and Modeling, U.S. Environmental Protection Agency, Research Triangle Park, Durham, NC 27711, USA;

**Keywords:** response model, ozone, PM_2.5_, air quality management

## Abstract

Reducing PM_2.5_ and ozone concentrations is important to protect human health and the environment. Chemical transport models, such as the Community Multiscale Air Quality (CMAQ) model, are valuable tools for exploring policy options for improving air quality but are computationally expensive. Here, we statistically fit an efficient polynomial function in a response surface model (pf-RSM) to CMAQ simulations over the eastern U.S. for January and July 2016. The pf-RSM predictions were evaluated using out-of-sample CMAQ simulations and used to examine the nonlinear response of air quality to emission changes. Predictions of the pf-RSM are in good agreement with the out-of-sample CMAQ simulations, with some exceptions for cases with anthropogenic emission reductions approaching 100%. NO_X_ emission reductions were more effective for reducing PM_2.5_ and ozone concentrations than SO_2_, NH_3_, or traditional VOC emission reductions. NH_3_ emission reductions effectively reduced nitrate concentrations in January but increased secondary organic aerosol (SOA) concentrations in July. More work is needed on SOA formation under conditions of low NH_3_ emissions to verify the responses of SOA to NH_3_ emission changes predicted here. Overall, the pf-RSM performs well in the eastern U.S., but next-generation RSMs based on deep learning may be needed to meet the computational requirements of typical regulatory applications.

## 1. Introduction

PM_2.5_ and ozone air pollution lead to harmful effects on human health and the environment [1,2]. Air quality management plans are developed to reduce these criteria pollutant concentrations to meet National Ambient Air Quality Standards (NAAQS) in the U.S. [3–5]. Air quality modeling with comprehensive chemical transport models (CTMs) contributes key information to air quality planning by providing concentration predictions for baseline and policy-relevant emission-control conditions [6].

Air quality modeling is important for effective air quality management because the response of PM_2.5_ and ozone to precursor emission changes is nonlinear and depends on hundreds of chemical reactions. For instance, ozone concentrations decrease in response to NO_x_ emission reductions when NO_x_ is the limiting precursor for oxidant formation but increase under NO_x_-saturated conditions, where NO_x_ inhibits oxidant production [7]. PM_2.5_ nitrate can also increase or decrease in response to NO_x_ emission reductions depending on oxidant levels and other factors, such as aerosol pH, temperature, and relative humidity [8–12]. Previous studies have individually reported seasonal variations in the nonlinear response of ozone and PM_2.5_ to NO_x_ emission reductions in the U.S. for retrospective periods [13,14]. However, information is limited on the seasonal variation in the simultaneous response in ozone and PM_2.5_ and its components for multiple precursors under recent conditions in the U.S. Consideration of relatively recent conditions is important because NO_x_ emissions declined by 57% and SO_2_ emissions by 85% between 2000 and 2017 (https://gispub.epa.gov/neireport/2017/, accessed on 11 August 2021).

A challenge in using CTMs to explore hypothetical policy options is computational expense. The runtime for CTM simulations (days to weeks) typically prevents direct modeling of the dozens to hundreds of possible emission scenarios that may be of interest to policymakers [15–17]. As a result, researchers have developed computationally efficient approaches that approximate CTM capabilities [18–27]. Of these approaches, response surface models (RSMs) are unique in their ability to simulate the nonlinear response of ozone and PM_2.5_ over wide ranges of precursor emission changes. RSMs are developed by statistically modeling the results of multiple CTM simulations with a set of explanatory variables based on the emission inputs for each simulation compared to a baseline simulation. After fitting, RSMs can provide predictions of the air quality response to emission changes in near real-time. RSMs have been developed to provide the air quality response to emission changes rather than changes in other variables (e.g., meteorology) because pollutant emissions are the key modifiable factors in air quality management applications.

Early-generation RSMs required large numbers of CTM simulations to produce a statistical fit to capture the complex pollutant responses simulated by CTMs [21,23,28,29]. To reduce the number of simulations for cases with multiple regions and emission control factors, the extended RSM (ERSM) technique was developed [20,24]. Next, using prior knowledge from ERSM results, a polynomial function-RSM (pf-RSM) approach was developed that further reduced the required number of CTM simulations by using polynomial functions to capture the nonlinear response of PM_2.5_ and ozone to emission changes [19]. Recently, the DeepRSM method [22] has been developed to efficiently calculate the polynomial function coefficients using a convolutional neural network trained with chemical indicators [30]. Much of the development of RSM technology has happened through the ABaCAS (Air Benefit and Cost and Attainment Assessment System, http://www.abacas-dss.com, accessed on 11 August 2021) project, and applications of recent RSMs have been limited to regions in Asia. The performance and applicability of recent RSM methods for the U.S. and other regions needs to be established for these approaches to gain broader use.

In this study, we fit a one-region pf-RSM [19] to CTM simulations over the eastern U.S. for January and July of 2016. We characterize the performance of the pf-RSM using 30 out-of-sample (OOS) CTM simulations. We also provide insights on the nonlinear response of air pollution in the eastern U.S. to anthropogenic emission reductions in winter and summer. We focus here on the response of maximum daily 8 h average (MDA8) of ozone, PM_2.5_, nitrate, sulfate, and organic matter (OM) concentrations to emission changes of NO_x_, SO_2_, NH_3_, and traditional volatile organic compounds (VOCs). The pf-RSM also simulates the response of PM_2.5_ concentrations to primary PM_2.5_ emissions and is available for download.

## 2. Methods

### 2.1. Base-Case CTM Simulation

CTM simulations were performed for January and July 2016 with version 5.3.1 of the Community Multiscale Air Quality (CMAQ; https://zenodo.org/record/3585898#.YRXGeEARWUk, accessed on 11 August 2021) model on a domain covering the eastern U.S. with 12 km grid spacing and 35 vertical layers. January and July were selected to be representative of winter and summer conditions, respectively. Gas-phase chemistry was parameterized according to the Carbon Bond 2006 mechanism (CB6r3) [31], the deposition was modeled with the M3DRY parameterization, and aerosol processes were parameterized with the AERO7 module using the non-volatile treatment for primary organic aerosol [32,33]. Chemical boundary conditions were developed from a CMAQ simulation on a larger domain that used boundary conditions from a hemispheric CMAQ simulation [34]. The starting point for the modeled anthropogenic emissions was version 2 of the 2014 National Emissions Inventory (NEI); however, many inventory sectors were updated to represent the year 2016 through the incorporation of 2016-specific state and local data along with nationally-applied adjustment methods [35]. Emissions of anthropogenic precursors for secondary organic aerosol (SOA) [36] were not added to the simulation beyond what was captured in the NEI. Therefore, VOC impacts discussed below are due to traditional VOCs alone. Emissions of biogenic compounds were modeled with the Biogenic Emission Inventory System (BEIS) [37], and emissions of sea-spray aerosol [38] were simulated online within CMAQ using 2016 meteorology. Meteorological fields were developed from a simulation with version 3.8 of the Weather Research and Forecasting model as described elsewhere [39].

Model performance for the base-case CTM simulation was evaluated by comparison with available monitoring data for PM_2.5_, PM_2.5_ components, and MDA8 ozone (Supplementary Text S1, Supplementary Table S1) (Supplementary Materials, Tables S1—S4). The model performance statistics are generally within ranges reported in previous applications [40,41] and support the modeling here. However, overpredictions of PM_2.5_ organic carbon concentrations were evident in January, possibly due to issues with emissions or meteorology as well as gas-particle partitioning of primary organic aerosol. The performance results in Supplementary Table S1 should therefore be considered in interpreting the RSM predictions. Model performance results here are qualitatively consistent with Appel et al. [42], although statistics are calculated for different periods and are not directly comparable across studies.

### 2.2. Sensitivity CTM Simulations and pf-RSM Development

In addition to the base-case simulation, 22 simulations were conducted for model fitting with domain-wide changes in U.S. anthropogenic emissions of NO_x_, SO_2_, VOC, NH_3_, and primary PM_2.5_ (see Supplementary Table S3). For 19 of these simulations, emission changes were specified based on Hammersley sampling [43] of emission control ratios between 0 and 1.2 (base case = 1.0). Additionally, one simulation was conducted with 100% reductions in U.S. anthropogenic emissions of NO_x_, SO_2_, NH_3_, and VOCs, and two simulations were conducted with 50% and 100% reductions in primary PM_2.5_ emissions. Emission-perturbation simulations were implemented in CMAQ using the Detailed Emissions Scaling, Isolation, and Diagnostic (DESID) module [44]. Version 2.5 of the RSM-VAT software was used to implement the Hammersley sampling and generate the emission-control interface files for DESID.

Polynomial functions were fit in each grid cell to provide the nonlinear response of monthly average PM_2.5_, PM_2.5_ components, and MDA8 ozone to changes in NO_x_, SO_2_, VOC, NH_3_, and primary PM_2.5_ emissions across the domain. The following optimized polynomial functions developed in the previous work [19,45] were fit using results of the emission reduction simulations in Supplementary Table S3:

(1) $$\Delta {\text{PM}}_{2.5,\text{spc}}={\text{X}}_{1}\;\Delta {\text{E}}_{\text{NOX}}+{\text{X}}_{2}\;\Delta {\text{E}}_{\text{SO}2}+{\text{X}}_{3}\;\Delta {\text{E}}_{\text{NH}3}+{\text{X}}_{4}\;\Delta {\text{E}}_{\text{VOC}}+{\text{X}}_{5}\;\Delta {\text{E}}_{{\text{NOX}}^{2}}+{\text{X}}_{6}\;\Delta {\text{E}}_{\text{SO}2^{2}}+{\text{X}}_{7}\;\Delta {\text{E}}_{\text{NH}3^{2}}+{\text{X}}_{8}\;\Delta {\text{E}}_{\text{NOX}}\;\Delta {\text{E}}_{\text{VOC}}+{\text{X}}_{9}\;\Delta {\text{E}}_{{\text{NOx}}^{3}}+{\text{X}}_{10}\;\Delta {\text{E}}_{{\text{NOx}}^{2}}\;\Delta {\text{E}}_{\text{VOC}}+{\text{X}}_{11}\;\Delta {\text{E}}_{{\text{NOx}}^{2}}\;\Delta {\text{E}}_{\text{SO}2}+{\text{X}}_{12}\;\Delta {\text{E}}_{{\text{NOx}}^{2}}\;\Delta {\text{E}}_{\text{NH}3}$$

(2) $$\Delta \text{MDA}8{\text{ O}}_{3}={\text{Y}}_{1}\;\Delta {\text{E}}_{\text{NOX}}+{\text{Y}}_{2}\;\Delta {\text{E}}_{\text{SO}2}+{\text{Y}}_{3}\;\Delta {\text{E}}_{\text{NH}3}+{\text{Y}}_{4}\;\Delta {\text{E}}_{\text{VOC}}+{\text{Y}}_{5}\;\Delta {\text{E}}_{{\text{NOX}}^{2}}+{\text{Y}}_{6}\;\Delta {\text{E}}_{\text{NOX}}\;\Delta {\text{E}}_{\text{NH}3}+{\text{Y}}_{7}\;\Delta {\text{E}}_{{\text{NOX}}^{2}}\;\Delta {\text{E}}_{\text{NH}3}$$

where ΔPM_2.5,spc_ refers to the change from the base case in the concentration of PM_2.5_, PM_2.5_ nitrate, PM_2.5_ sulfate, or PM_2.5_ organic matter; ΔE refers to the ratio of the difference in emissions between the base and emission perturbation cases to the base emissions (i.e., ΔE_i_ = (E_i_ − E_Base_)/E_Base_); and X_1–12_ and Y_1–7_ are the polynomial coefficients determined by least-squares error fitting.

The pf-RSM was evaluated by comparison with 30 OOS CMAQ simulations that were not used in model fitting (Table S4). The OOS simulations included 10 simulations based on Hammersley sampling and 20 simulations corresponding to 20%, 40%, 60%, 80%, and 100% reductions in U.S. anthropogenic NO_x_, SO_2_, NH_3_, and VOC emissions.

The strength of the RSM is the ability to conduct interactive exploratory analyses on air quality impacts for an unconstrainted number of emission cases using the RSM-VAT software. Below, we focus on comparisons of pf-RSM and CMAQ OOS predictions to demonstrate the performance of the pf-RSM. We also use the OOS simulations to illustrate features of the nonlinear response of ozone and PM_2.5_ and its components in the eastern U.S. in winter and summer. The RSM-VAT software is available with cases preloaded for exploration of additional scenarios.

## 3. Results

Predictions of the pf-RSM are compared with CMAQ results for the 30 OOS runs in this section. The comparisons illustrate the performance of the pf-RSM as well as the nonlinear response of PM_2.5_ and MDA8 ozone to reductions in U.S. anthropogenic emissions. Average monitored concentrations of PM_2.5_, PM_2.5_ components, and MDA8 ozone in the region are provided in Supplementary Table S1 and have been discussed in previous studies [14].

### 3.1. January

**Figure 1.**
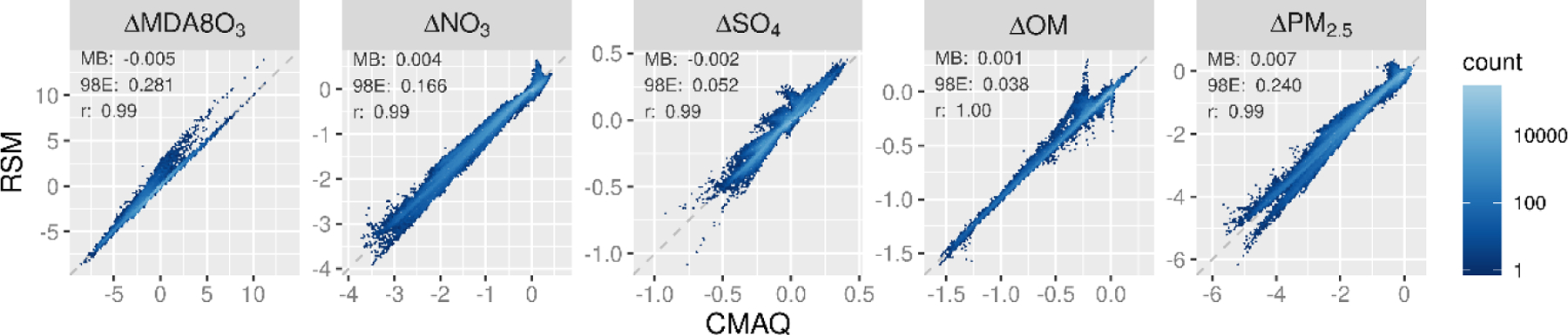
Comparison of changes in mean January concentrations predicted by the pf-RSM and 30 OOS CMAQ simulations. Units: ppb for MDA8 ozone and μg m^−3^ for PM_2.5_ and its components.

The air quality response to emission changes predicted by the pf-RSM and CMAQ are compared for the 30 OOS cases for five species in Figure 1. Overall, there is an excellent correlation and slight bias between the pf-RSM and CMAQ results. However, some distinct features are evident in the scatterplots due to the specific conditions of the individual OOS simulations. For instance, most points in the MDA8 ozone panel are close to the one-to-one line, but a cluster points above the line indicates some overpredictions by the pf-RSM. These points are associated with the case of 100% NO_x_ emission reductions (see Supplementary Figure S1 for scatterplots faceted by OOS case) (Supplementary Materials, Figures S1–S10). Challenges in simulating pollutant response for cases with extreme emission reductions have been reported in the past and suggest a need for including additional CTM simulations in pf-RSM fitting for applications where deep emission reductions may be relevant [19].

pf-RSM predictions of the nitrate response to emission changes also agree well with the OOS CMAQ results, despite some overpredictions of nitrate concentration increases (i.e., disbenefits). These overestimates are associated with cases of SO_2_ emission reductions, where disbenefits are overpredicted by up to ~0.4 μg m^−3^ for the case of 100% reduction in anthropogenic SO_2_ emissions (Supplementary Figure S2). For pf-RSM predictions of the sulfate response, the largest deviations from the one-to-one line in Figure 1 are associated with OOS Run 4 and 5 based on Hammersley sampling of emissions (Supplementary Figure S3). These runs included deep reductions in NO_x_, VOC, NH_3_, and SO_2_ emissions (Supplementary Table S4).

For the OM concentration response, there is good agreement between the pf-RSM and CMAQ predictions in general. However, the pf-RSM predicts some disbenefits for the 100% NO_x_ emission reduction case (Supplementary Figure S4) that were not predicted by CMAQ. For the total PM_2.5_ concentration response, the scatterplot has a forked shape for concentration decreases larger than 4 μg m^−3^. This pattern results from the combination of underpredictions in response to the pf-RSM in the 100% NH_3_ emission reduction case and overpredictions in a case with large NO_x_ reductions (i.e., Run 5 with 97% NO_x_, 90% SO_2_, 24% NH_3_, and 81% VOC emission reductions) (Supplementary Figure S5). This behavior further demonstrates that deviations of the pf-RSM from CMAQ may be relatively large in cases with large emission reductions, and model fitting could be improved by including additional simulations for marginal emission cases. Since emission reductions approaching 100% are uncommon in typical regulatory applications, pf-RSM performance issues for these marginal cases may be of limited concern in many applications.

**Figure 2.**
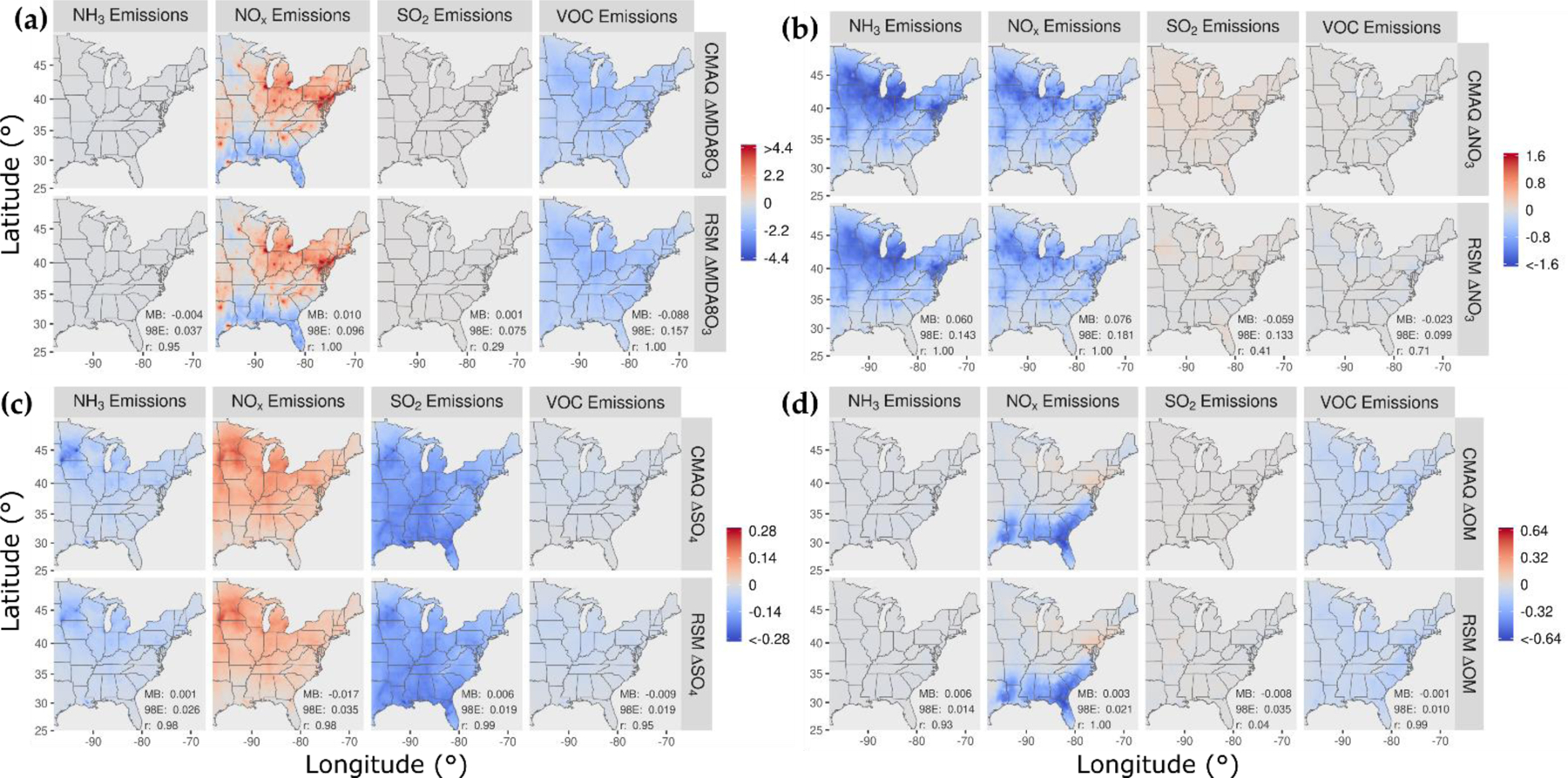
Comparison of the change in average concentration in January 2016 for the pf-RSM and CMAQ for a 60% reduction in anthropogenic emissions of NH_3_, NO_x_, SO_2_, and VOC: (**a**) MDA8 ozone, (**b**) PM_2.5_ nitrate, (**c**) PM_2.5_ sulfate, and (**d**) PM_2.5_ OM. Units: ppb for MDA8 ozone and μg m^−3^ for PM_2.5_ components. Statistics for pf-RSM and CMAQ comparison: MB: mean bias; 98E: 98th percentile of error; r: Pearson correlation coefficient.

In Figure 2, the spatial patterns of concentration responses in January 2016 are compared for CMAQ and the pf-RSM for MDA8 ozone, nitrate, sulfate, and OM for 60% reductions in NH_3_, NO_x_, SO_2_, and VOC emissions. The response patterns for the pf-RSM and CMAQ are in good agreement in all cases. NO_x_ emission reductions in January lead to increases in MDA8 ozone over much of the domain (Figure 2a), especially in northern and urban areas (e.g., the mean/max concentration increase above 37° N is 0.8/7.1 ppb for CMAQ). The ozone disbenefits for NO_x_ emission reductions are consistent with oxidant-limited conditions in NO_x_-rich areas in winter [14,46,47]. By contrast, MDA8 ozone concentrations decrease in response to NO_x_ emission reductions along the Gulf Coast (except Houston) and over Florida, likely due to the lower NO_x_ emissions and inflow of marine air. For VOC, the 60% emission reductions reduce MDA8 ozone concentrations broadly over the eastern U.S. (CMAQ mean reduction: 1.1 ppb).

The 60% reductions in NH_3_ and NO_x_ emissions reduce nitrate concentrations in the northern part of the domain and demonstrate the sensitivity of nitrate to both precursors there (Figure 2b). NH_3_ emission reductions in areas of elevated NH_3_ concentration [48] can reduce nitrate concentrations by increasing particle acidity (due to removal of the key atmospheric base, NH_3_) and thereby reducing the fraction of total nitrate in the particle phase [9,49,50]. NO_x_ emission reductions reduce nitrate through direct removal of the nitrate precursor, which outweighs the effect of increased NO_x_-to-nitrate conversion efficiency from increased ozone/oxidant concentrations. For reducing nitrate in January, NH_3_ emission reductions (mean nitrate reduction: 0.62 μg m^−3^) are more effective than NO_x_ emission reductions (mean nitrate reduction: 0.46 μg m^−3^). SO_2_ and VOC emission reductions have relatively small influence on nitrate, with some nitrate disbenefits for SO_2_ reductions in the northern part of the domain (likely due to the influence of reduced acidity from lower sulfate on partitioning of total nitrate to the particle phase).

The 60% reductions in NH_3_ emissions lead to small decreases in sulfate concentrations in the northern part of the domain (Figure 2c). Since sulfate is essentially nonvolatile under atmospheric conditions, NH_3_ levels do not affect gas-particle partitioning of sulfate as they do for semi-volatile nitrate. However, in-cloud sulfate production is sensitive to cloud pH, and the reductions in NH_3_ concentrations could reduce cloud pH and thereby lower the rate of S(IV) to S(VI) conversion (e.g., due to ozone pathways, [51]). Shah et al. [52] reported that in-cloud oxidation was responsible for about 65% of the conversion of SO_2_ to sulfate in the eastern U.S. in winter 2015. NO_x_ emission reductions lead to increases in sulfate in the northern part of the domain due to the increases in ozone and other oxidants that promote the conversion of SO_2_ to sulfate. SO_2_ emission reductions reduce sulfate throughout the domain by removing the sulfate precursor, and VOC reductions reduce sulfate by a small amount by reducing ozone and other oxidants.

The response of OM concentrations to 60% emission reductions is shown in Figure 2d. NO_x_ emission reductions reduce OM concentrations throughout the southeast but increase concentrations slightly in the northeast. In winter, both monoterpene [53] and aromatic [54] oxidation contribute to SOA concentrations. Reductions in NO_x_ emissions lower the concentrations of monoterpene nitrate precursors in the southeast [55] and reduce the oxidation of monoterpenes in areas where ozone and OH decrease [56]. In the northeast, the increases in OM concentrations with NO_x_ emission reductions are consistent with more efficient conversion of SOA precursors to OM due to increased oxidant (e.g., ozone) concentrations. In addition, reducing NO_x_ shifts aromatic oxidation to higher-yield SOA pathways [54].

**Figure 3.**
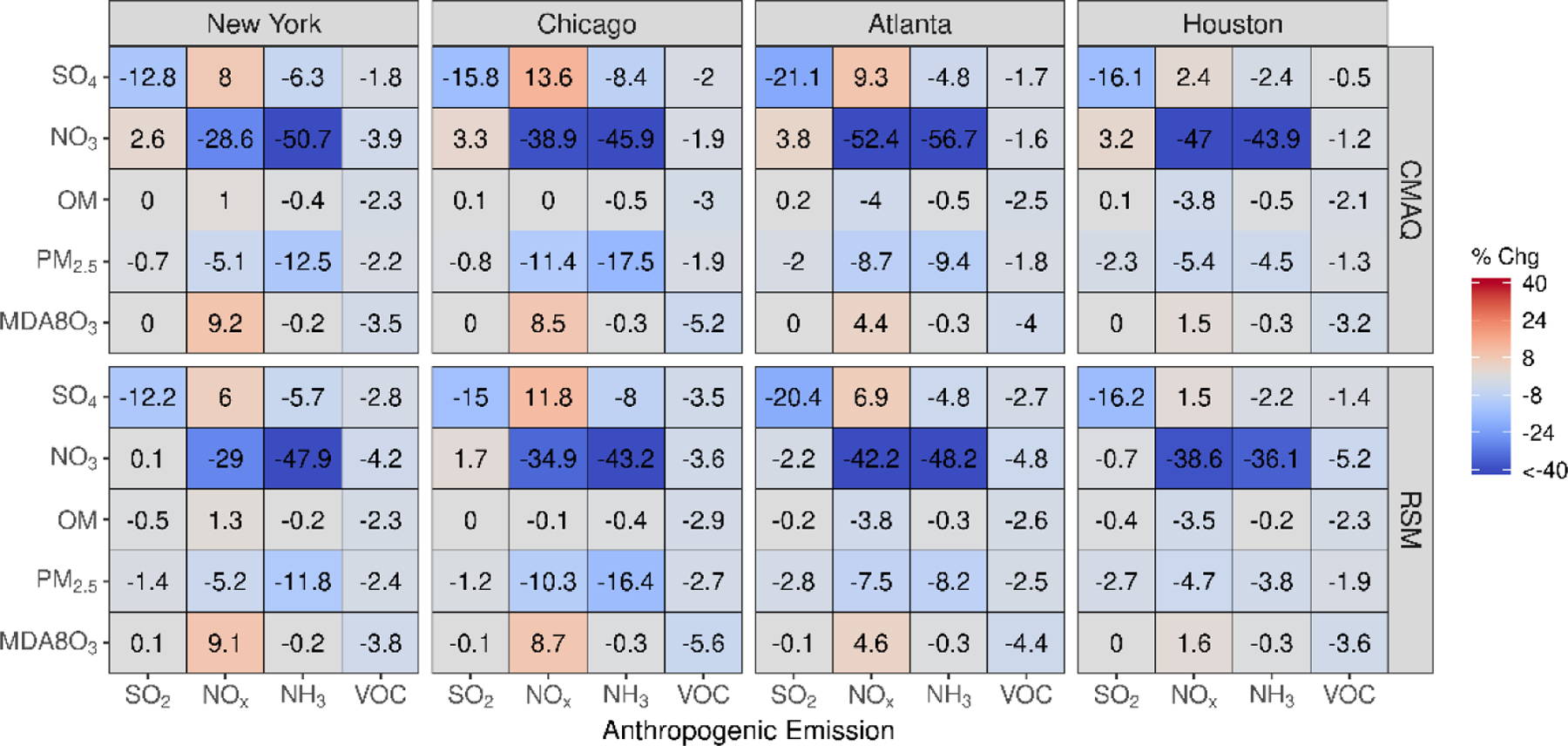
Comparison of the percent change in pollutant concentrations for four urban CBSAs during January 2016 as predicted by the pf-RSM and CMAQ. Units: ppb for MDA8 ozone and μg m^−3^ for PM_2.5_ and its components.

In Figure 3, the percent change in concentration is shown for a 60% reduction in emissions for grid cells in four urban core-based statistical areas (CBSAs). Good agreement exists between CMAQ and pf-RSM predictions in the CBSAs. In the CMAQ simulations, reductions in anthropogenic NO_x_ emissions increase MDA8 ozone by 9% (about 2.5 ppb) in NY and Chicago and a smaller amount in Atlanta (4%, 1.4 ppb) and Houston (2%, 0.4 ppb). NO_x_ emission reductions reduce nitrate by 29% (0.6 μg m^−3^) in NY, 39% in Chicago (1.2 μg m^−3^), 52% in Atlanta (0.6 μg m^−3^), and 47% in Houston (0.3 μg m^−3^). Decreases in NH_3_ emissions also reduce nitrate in the CBSAs: 51% (1.1 μg m^−3^) in NY, 46% in Chicago (1.4 μg m^−3^), 57% in Atlanta (0.6 μg m^−3^), and 44% in Houston (0.3 μg m^−3^). SO_2_ emission reductions reduce sulfate concentrations by 0.11 to 0.18 μg m^−3^ but increase nitrate concentrations by 0.02 to 0.1 μg m^−3^ in the CMAQ simulations. In contrast to the slight nitrate disbenefits predicted by CMAQ, the pf-RSM predicted small nitrate reductions in Atlanta and Houston for the SO_2_ emission reductions. NH_3_ emission reductions produce the greatest reductions in PM_2.5_ concentrations in January in all CBSAs except Houston.

**Figure 4.**
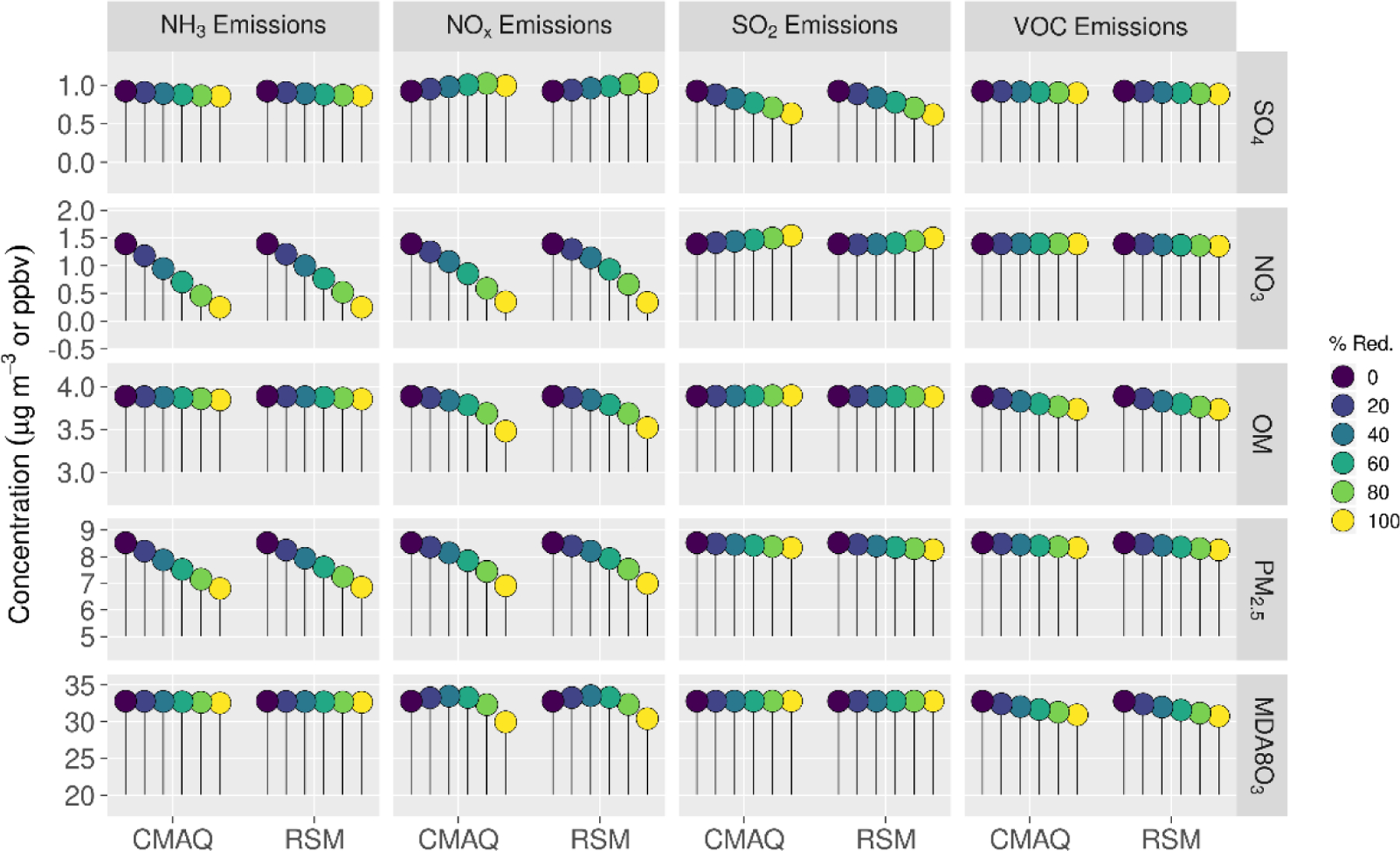
Comparison of the mean absolute concentrations predicted by the pf-RSM and CMAQ over all CBSAs in the domain during January 2016 for U.S. anthropogenic emission changes from 0 to 100%. Units: ppbv for MDA8 ozone and μg m^−3^ for PM_2.5_ and its components.

Comparisons of mean absolute concentrations predicted by the pf-RSM and CMAQ over all CBSAs in the domain for 0% to 100% emission reductions in January 2016 are provided in Figure 4. The predictions discussed above for the 60% emission reductions are generally reflective of the model response indicated in Figure 4, although the disbenefits in MDA8 ozone for 60% NO_x_ emission reductions transition to benefits for larger reductions. For instance, MDA8 ozone increased over the CBSAs by 0.7 ppb for the 40% NO_x_ emission reduction but decreased by 2.8 ppb for the 100% NO_x_ reduction in the CMAQ simulations.

The trend of increasing sulfate with decreasing NO_x_ emissions (Figure 4) is consistent with Shah et al. [52], who reported that the SO_2_-to-sulfate conversion efficiency increased from 0.11 to 0.18 in winter in the eastern U.S. due to emission reductions during the 2007–2015 period. For CMAQ predictions, the trend of increasing sulfate with decreasing NO_x_ emissions reverses for NO_x_ reductions greater than about 80% but continues for the pf-RSM. pf-RSM performance could be improved for this case by including additional simulations in model fitting [19], although there could also be limitations in the polynomial functions for representing the entire range of concentration response. Nevertheless, the pf-RSM generally captures the CMAQ responses across species and emission changes, including the overall response in PM_2.5_ concentrations. CMAQ predictions of changes in mean PM_2.5_ concentrations over the CBSAs for 100% reductions in anthropogenic emissions are −1.72 μg m^−3^ (−20%, NH_3_ emissions); −1.61 μg m^−3^ (−19%, NO_x_ emissions); and −0.19 μg m^−3^ (−2.2%, SO_2_ and VOC emissions). The PM_2.5_ concentration reductions associated with 100% NO_x_ emission reductions overcome a 0.07 μg m^−3^ sulfate disbenefit, and the PM_2.5_ concentration reductions for 100% SO_2_ emission reductions overcome a 0.15 μg m^−3^ nitrate disbenefit (about 50% of the sulfate reduction in that case).

### 3.2. July

**Figure 5.**
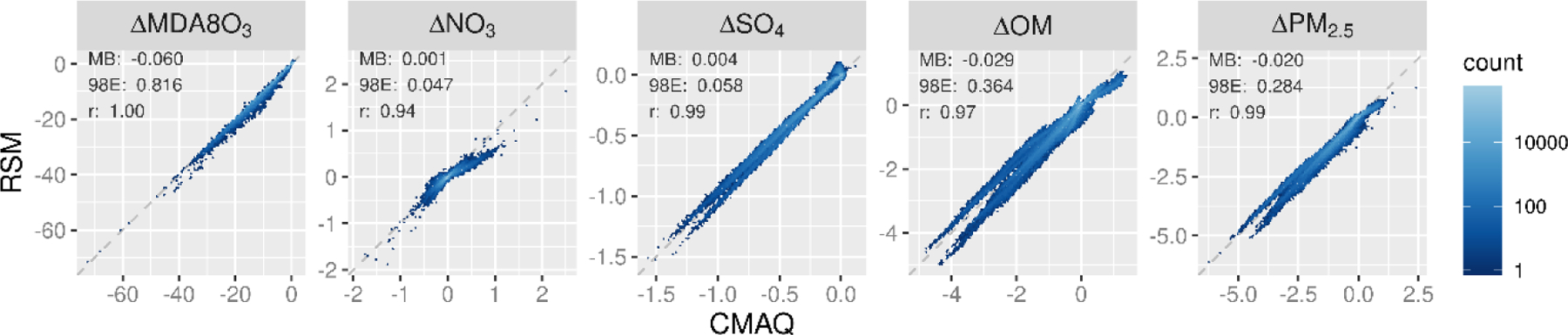
Comparison of changes in mean July concentrations predicted by the pf-RSM and 30 OOS CMAQ simulations. Units: ppb for MDA8 ozone and μg m^−3^ for PM_2.5_ and its components.

The mean concentration responses in July 2016 predicted by the pf-RSM and CMAQ are compared for the 30 OOS cases in Figure 5. The pf-RSM predictions for MDA8 ozone agree well with CMAQ results across the full set of OOS simulations, even for the case of 100% NO_x_ emission reductions with large (>30 ppb) MDA8 ozone decreases (Supplementary Figure S6). Nitrate responses are also in general agreement for the pf-RSM and CMAQ, although the pf-RSM tends to underestimate the magnitude of the disbenefits predicted by CMAQ. These underestimates are associated with the 100% SO_2_ emission reduction simulation (Supplementary Figure S7).

For the sulfate response, a forked pattern exists in the scatterplot for concentration decreases larger than 1 μg m^−3^. This pattern results from pf-RSM underestimates of the CMAQ response for the 100% SO_2_ emission reduction case and overestimates for the Run 5 case. The forked pattern for the OM response is due to pf-RSM response overestimates for the 80% and 100% NO_x_ emission reduction cases and slight underestimates for the Run 5 case. Moreover, the OM disbenefits predicted by CMAQ were underestimated by the pf-RSM for the 100% NH_3_ emission reduction case (Supplementary Figure S9). The forked pattern in the scatterplot for the PM_2.5_ response as well as the pf-RSM underestimate of PM_2.5_ disbenefits follows the behavior for OM (i.e., PM_2.5_ responses are overestimated for the 100% NO_x_ emission reductions and disbenefits are underestimated for 100% NH_3_ emission reductions, Supplementary Figure S10).

**Figure 6.**
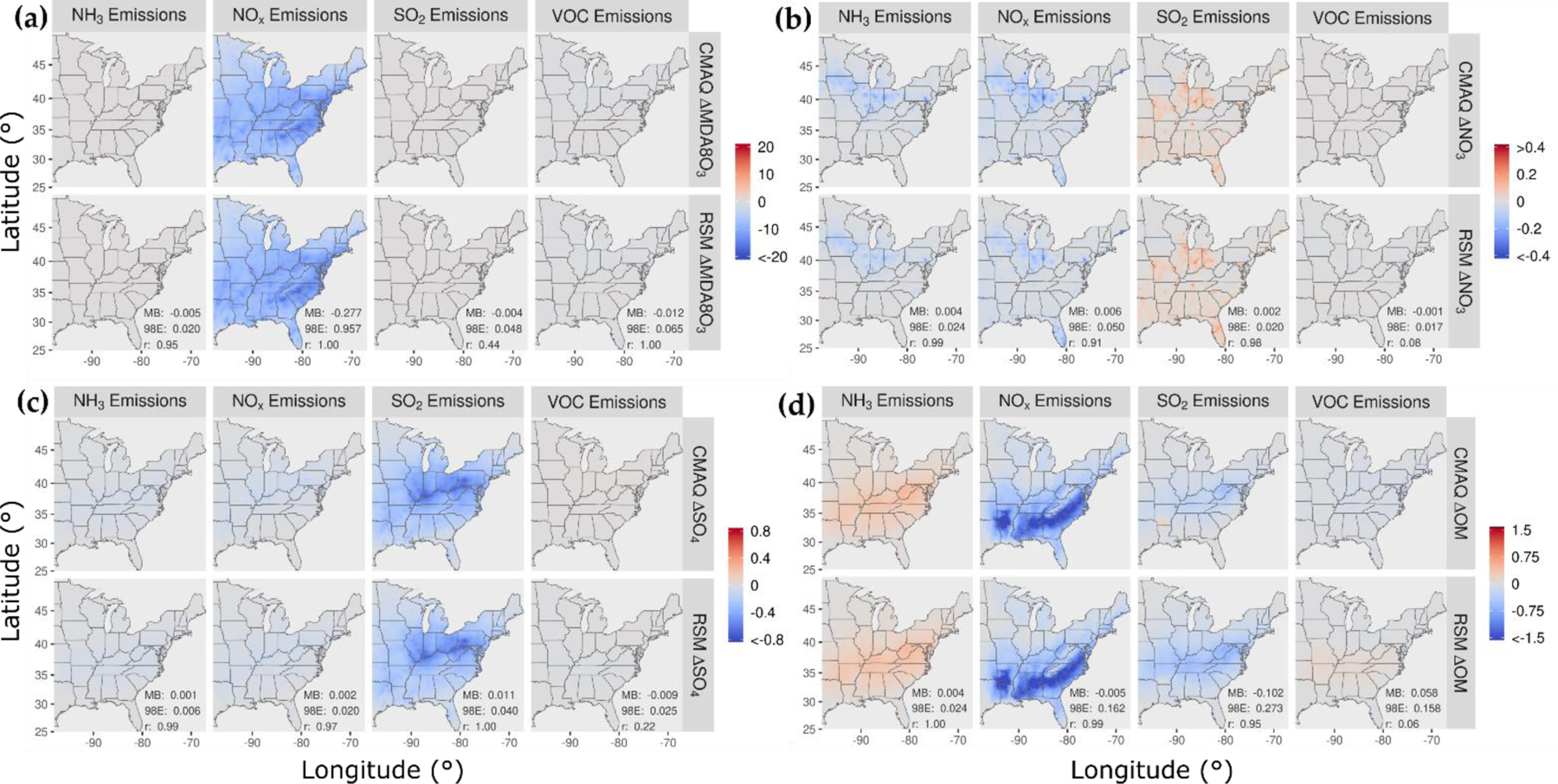
Comparison of the change in average concentration in July 2016 for the pf-RSM and CMAQ for a 60% reduction in anthropogenic emissions of NH_3_, NO_x_, SO_2_, and VOC: (**a**) MDA8 ozone, (**b**) PM_2.5_ nitrate, (**c**) PM_2.5_ sulfate, and (**d**) PM_2.5_ OM. Units: ppb for MDA8 ozone and μg m^−3^ for PM_2.5_ components. Statistics for pf-RSM and CMAQ comparison: MB: mean bias; 98E: 98th percentile of error; r: Pearson correlation coefficient.

In Figure 6, the spatial patterns of concentration responses in July 2016 are compared for CMAQ and the pf-RSM for MDA8 ozone, nitrate, sulfate, and OM for 60% reductions in NH_3_, NO_x_, SO_2_, and VOC emissions. The pf-RSM and CMAQ response patterns are generally in good agreement across cases. For MDA8 ozone, the 60% NO_x_ emission reductions lead to large ozone decreases (mean: 7 ppb) throughout the eastern U.S. (Figure 6a), in contrast to the ozone increases predicted in January in northern and urban areas. Emission reductions for other species have a relatively small effect on MDA8 ozone. The 60% reductions in NO_x_ and NH_3_ emissions reduce nitrate concentrations through a band of cells from Iowa to Pennsylvania, and NO_x_ emissions reductions also reduce nitrate in parts of Florida (Figure 6b). The SO_2_ emission reductions cause increases in nitrate concentrations in some areas, likely due to chemical feedbacks of acidity on gas-particle partitioning of total nitrate. NO_x_ and NH_3_ emission reductions lead to small sulfate decreases due to the influence of NO_x_ on oxidant abundance and NH_3_ on cloud pH. SO_2_ reductions reduce sulfate in the Ohio Valley where large SO_2_ sources are located (Figure 6c).

**Figure 7.**
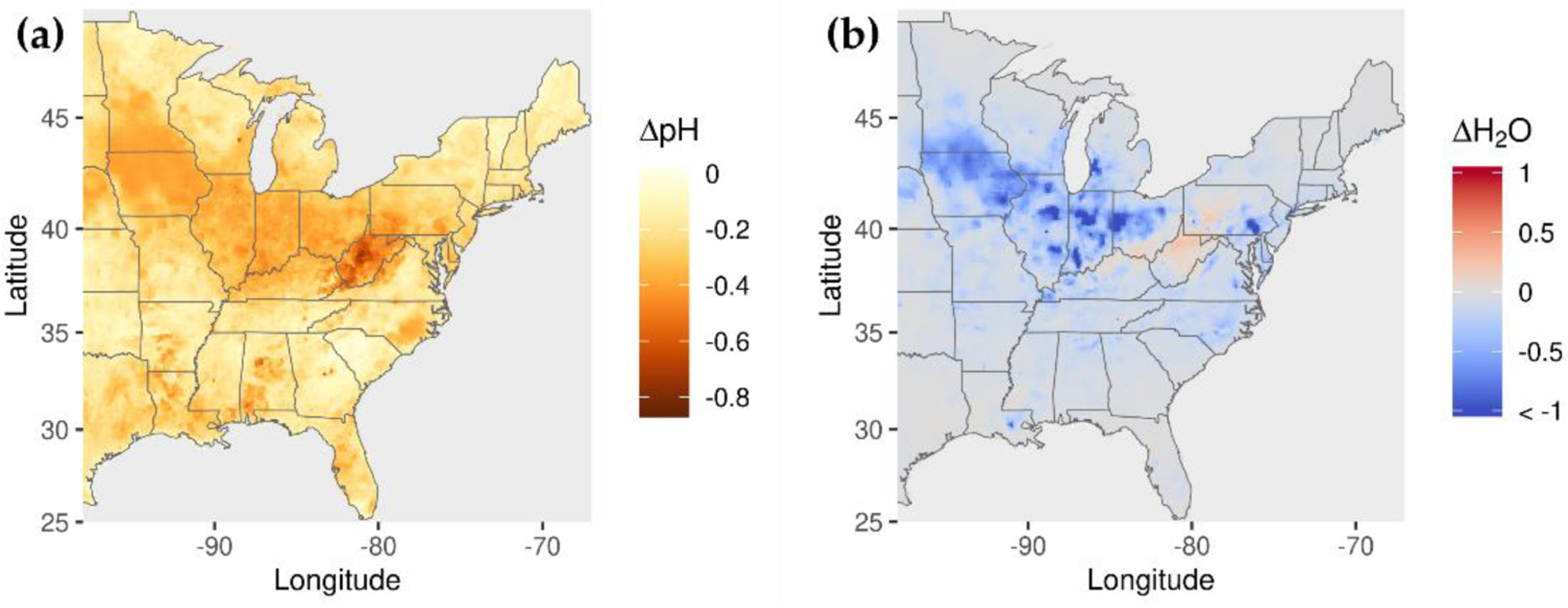
Change in (**a**) pH and (**b**) fine-particle water concentration for 60% reduction in NH_3_ emissions.

The 60% reductions in NH_3_ emissions increase OM concentrations along a latitude band between 32 N and 42 N. This behavior appears to be associated with biogenic SOA formation from acid-catalyzed uptake of isoprene epoxydiols (IEPOX) and subsequent in-particle reaction involving nucleophile addition to the parent hydrocarbon. This SOA formation pathway is enhanced under conditions of greater acidity and increased nucleophile (water and sulfate) concentration (i.e., eqn. 4 of Pye et al. [57]). The 60% reduction in NH_3_ emissions reduces pH by 0.23 on average (up to 0.84) (Figure 7a), which corresponds to a 75% increase in [H+] on average (up to 594%). These increases in acidity rather than changes in nucleophile concentrations explain the OM concentration increases, because sulfate concentrations decrease with decreasing NH_3_ emissions (Figure 6c) and aerosol water concentrations also generally decrease, except over a region around West Virginia with small (<9%) increases (Figure 7b).

The 60% reductions in NO_x_ emissions decrease OM concentrations in the southern U.S. where biogenic SOA is relatively high (Figure 6d). Previous work has found that reducing NO_x_ in the southeast U.S. in summer leads to substantial reductions in the organic nitrate fraction of OM and smaller changes for other OM contributors [55]. SO_2_ emission reductions reduce OM concentrations with a spatial pattern similar to that previously reported for the SO_2_ response of biogenic SOA formed via aerosol water chemistry [58]. As described above, SO_2_ emission reductions can reduce particle acidity and nucleophile concentrations and thereby lower biogenic SOA production. Anthropogenic VOC emission reductions lead to small OM concentration reductions in the CMAQ simulations, but the pf-RSM predicts small increases. This behavior did not occur in previous pf-RSM applications in China and should be investigated further in future studies.

**Figure 8.**
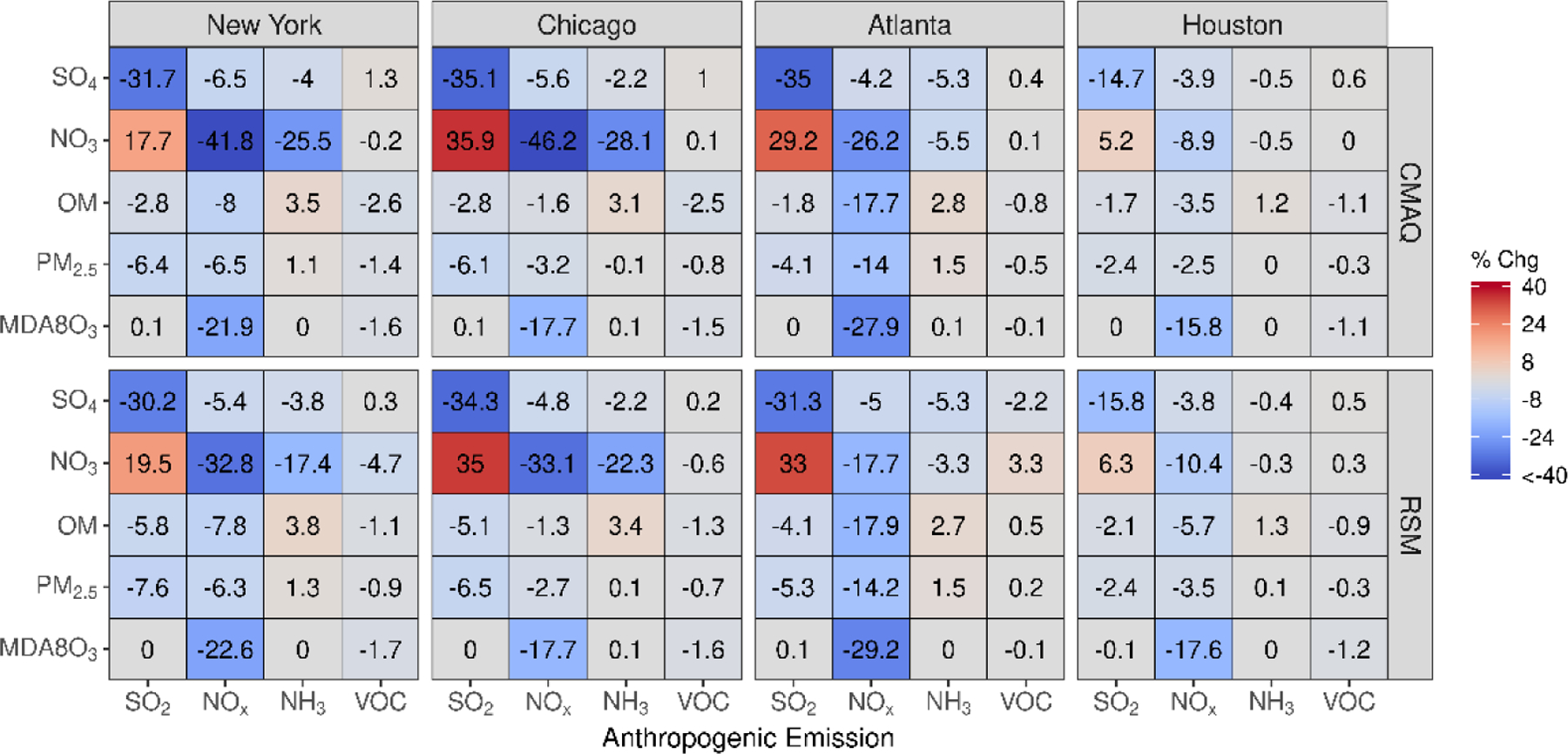
Comparison of the percent change in pollutant concentrations for four urban CBSAs during July 2016 as predicted by the pf-RSM and CMAQ. Units: ppb for MDA8 ozone and μg m^−3^ for PM_2.5_ and its components.

Good agreement exists between pf-RSM and CMAQ predictions of the percent change in concentrations over the four CBSAs in Figure 8 for 60% reductions in precursor emissions in July. In response to the SO_2_ emission reductions, sulfate concentrations decreased by 0.1 μg m^−3^ in Houston, 0.25 μg m^−3^ in Atlanta, 0.28 μg m^−3^ in NY, and 0.38 μg m^−3^ in Chicago. In contrast, the SO_2_ emission reductions caused some increases in nitrate concentrations, although the effect is small (0.02 μg m^−3^ in Houston and Atlanta, 0.03 μg m^−3^ in NY, and 0.09 μg m^−3^ in Chicago) due to the low nitrate concentrations in summer. In response to 60% NO_x_ emission reductions, MDA8 ozone concentrations decreased from 16% (5 ppb in Houston) to 28% (12 ppb in Atlanta) in the CMAQ simulations. NO_x_ emission reductions also caused decreases in OM concentrations in the CBSAs, with the greatest reduction in Atlanta (18%, 1.5 μg m^−3^), where biogenic SOA is prominent. NO_x_ emission reductions caused large percent reductions in nitrate concentrations (up to 36% in Chicago), but the absolute concentration changes are small (i.e., ≤0.12 μg m^−3^). NH_3_ emission reductions lead to decreases in nitrate concentrations and increases in OM concentrations in July. The OM increases are greater than the nitrate decreases in absolute terms (greater by 0.14 μg m^−3^ in NY, 0.03 μg m^−3^ in Chicago, 0.23 μg m^−3^ in Atlanta, and 0.02 μg m^−3^ in Houston). Anthropogenic VOC reductions have a small effect on concentrations in July due to the high levels of biogenic VOC, although this study did not consider intermediate and semi-volatile VOC beyond what is included in the NEI.

**Figure 9.**
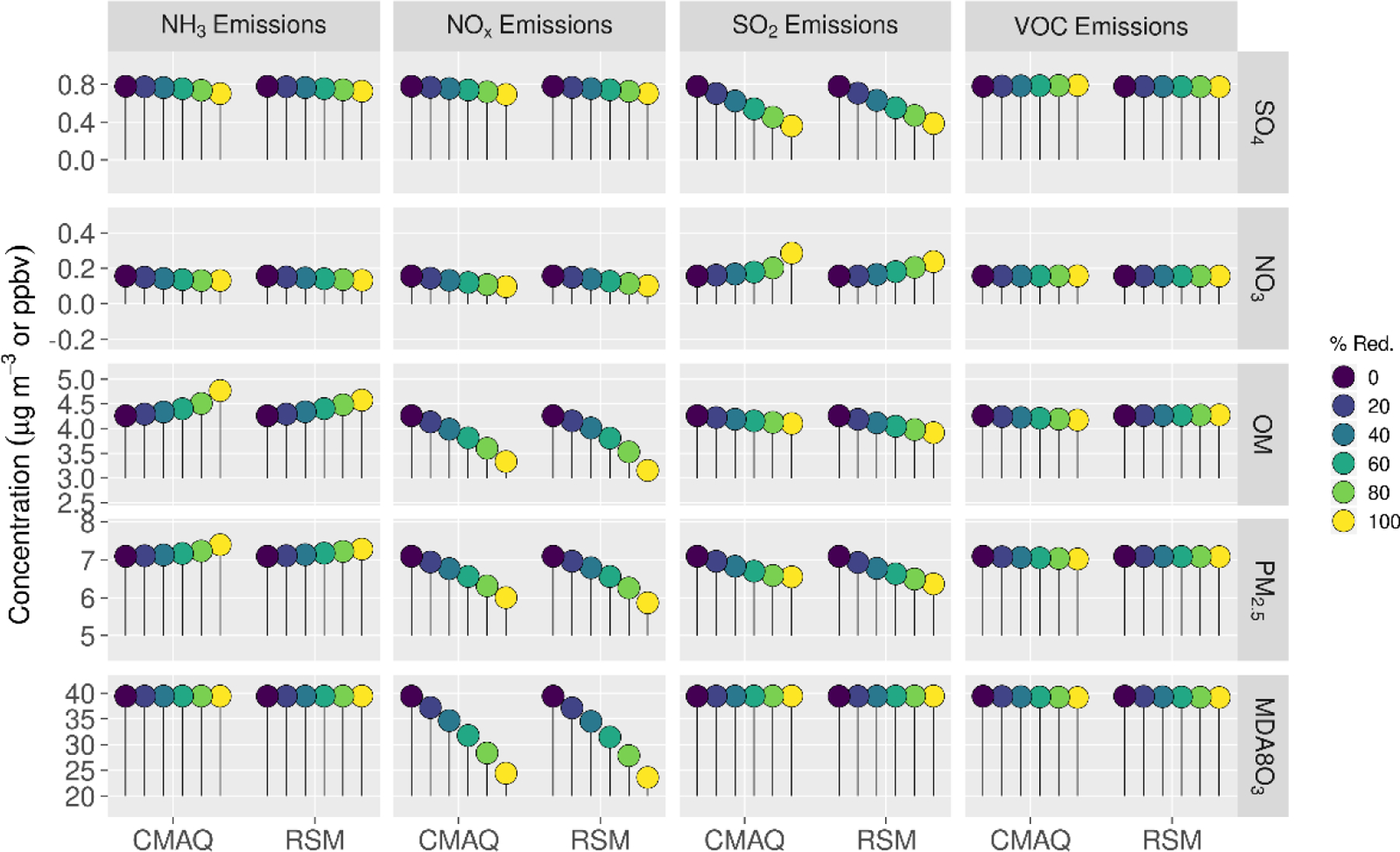
Comparison of the mean absolute concentrations predicted by the pf-RSM and CMAQ over all CBSAs in the domain during July 2016 for U.S. anthropogenic emission changes from 0 to 100%. Units: ppbv for MDA8 ozone and μg m^−3^ for PM_2.5_ and its components.

Comparisons of mean absolute concentrations predicted by the pf-RSM and CMAQ over all CBSAs in the domain for 0% to 100% emission reductions in July 2016 are shown in Figure 9. SO_2_ emission reductions reduce sulfate (up to 0.42 μg m^−3^) and OM (up to 0.16 μg m^−3^) concentrations but increase nitrate concentrations (up to 0.13 μg m^−3^). In contrast to January, NO_x_ emission reductions reduce MDA8 ozone and sulfate concentrations for all NO_x_ emission reduction levels due to the greater oxidant abundance in July. NO_x_ emission reductions also decrease OM concentrations (up to 0.93 μg m^−3^). NH_3_ emission reductions lead to increases in OM concentrations (up to 0.51 μg m^−3^), with increases growing nonlinearly with a decreasing emission level. As discussed above, the fact that OM concentration increases with decreasing NH_3_ emissions could be related to biogenic SOA formation associated with IEPOX uptake. Additional investigation of this SOA formation pathway under low NH_3_ and SO_2_ (and water content) conditions would be worthwhile. Riva et al. [59] reported that IEPOX organosulfates are highly viscous and likely to lead to phase separation under acidic conditions with low water content. Phase separation becomes more likely as sulfate decreases relative to IEPOX, resulting in increased diffusion barriers to further IEPOX uptake and SOA formation. Such behavior, which is not present in the base CMAQ model, could affect the sensitivity of OM to SO_2_ and NH_3_ emissions.

Predicted changes in PM_2.5_ concentrations for 100% reductions in anthropogenic emissions are −1.09 μg m^−3^ (−15.4%) (NO_x_ emissions); −0.54 μg m^−3^ (−7.67%) (SO_2_ emissions); −0.072 μg m^−3^ (−1.02%) (VOC emissions); and +0.30 μg m^−3^ (+4.3%) (NH_3_ emissions). The PM_2.5_ concentration decreases associated with 100% NO_x_ emission reductions include a small decrease in sulfate concentration of 0.09 μg m^−3^ (in contrast to January when sulfate concentrations increased with NO_x_ emission reductions). The PM_2.5_ concentration decreases for 100% SO_2_ emission reductions overcome a 0.13 μg m^−3^ increase in nitrate concentration (about 30% of the sulfate concentration decrease, 0.42 μg m^−3^). The predicted change in MDA8 ozone concentration for 100% reduction in NO_x_ emissions is −15.0 ppb (38%), and for 100% reduction in VOC emissions, it is 0.25 ppb.

## 4. Conclusions

Reducing PM_2.5_ and ozone concentrations is important to protect human health and the environment. CTMs are valuable tools for exploring policy options for improving air quality, but CTMs are computationally expensive and statistical models are therefore developed to approximate CTMs in some applications. Recent developments in RSM technology have reduced the number of CTM simulations needed for model fitting and provide an opportunity to evaluate RSM performance in the U.S.

Predictions of the pf-RSM developed here are in good agreement with OOS CMAQ simulations in the eastern U.S., with some exceptions for cases with anthropogenic emission reductions approaching 100%. These extreme conditions may have limited relevance in typical applications. Furthermore, previous work [19] suggests that performance can be improved in these cases by including additional simulations in pf-RSM fitting. Although the pf-RSM required fewer simulations for development than previous-generation RSMs, the one-region, five-emission factor pf-RSM still required about 20 CTM simulations. Therefore, computational expense would present challenges for developing pf-RSMs for multiple regions and emission sectors in typical applications. The recently developed DeepRSM approach [22], which requires fewer CTM simulations for fitting and improves performance compared with the pf-RSM, could facilitate the development of more complex RSMs in the future.

NO_x_ emission reductions were more effective for reducing PM_2.5_ concentrations than SO_2_, NH_3_, and traditional VOC emission reductions. In January, NO_x_ emission reductions decreased nitrate concentrations in the north and OM concentrations in the south. NO_x_ emission reductions did cause some disbenefits for sulfate concentrations in January, but the decreases in other PM_2.5_ components overcame the disbenefits. In July, NO_x_ emission reductions led to substantial decreases in OM concentrations by reducing biogenic SOA formation in the south. NH_3_ emission reductions were effective for reducing nitrate concentrations in January but increased OM concentrations in July. As a result, the effectiveness of NH_3_ emission reductions for reducing PM_2.5_ concentrations was less than for NO_x_ emission reductions overall. More work should be done to understand IEPOX SOA formation under conditions of low NH_3_ emissions to verify the OM responses predicted here. VOC emission reductions had a smaller effect on PM_2.5_ concentrations than NO_x_ and NH_3_ in part due to high levels of biogenic VOC in the eastern U.S. Moreover, our study did not include emissions of intermediate and semi-volatile VOC [36] beyond what is included in the NEI.

For MDA8 ozone concentrations, large reductions (>80%) in NO_x_ emissions are needed to avoid disbenefits in northern and urban areas in January. In July, all levels of NO_x_ emission reductions are effective for reducing MDA8 ozone due to the abundance of oxidants in summer. VOC emission reductions caused small decreases in MDA8 ozone concentrations in January and had little effect in July due to the high levels of biogenic VOC.

Since our study focused on the nonlinear response of pollutant concentrations, we did not discuss the influence of primary PM_2.5_ emissions on PM_2.5_ concentrations. However, PM_2.5_ concentrations are generally more responsive to reductions in primary PM_2.5_ emissions than the precursors for secondary PM_2.5_ discussed here. Some components of primary PM_2.5_ emissions (e.g., crustal cations) can also influence concentrations of secondary PM_2.5_ [60,61]. Large reductions in NO_x_ and SO_2_ emissions in the eastern U.S. in recent decades have reduced the concentrations of secondary inorganic aerosol and increased the importance of primary PM_2.5_ emissions and organic aerosol. Improved representations of the emissions and chemistry of organic aerosol are increasingly important in this context.

## Supplementary Material

Supplement1

## Data Availability

Publicly available datasets were analyzed in this study. This data can be found here: ftp://newftp.epa.gov/aqmg/cjang/RSM/RSM-VAT2.6/.
