# Supplementary material for: Predicting the Nonlinear Response of PM2.5 and Ozone to Precursor Emission Changes with a Response Surface Model
Source: Atmosphere (Basel). Author manuscript; Available in PMC 2022 Aug 14. (PMC8459679; doi:10.3390/atmos12081044)
Supplement: Supplement1 [file NIHMS1741146-supplement-Supplement1.pdf]

atmosphere

Article

Supporting Information for Predicting the Nonlinear Response of $PM_{2.5}$ and Ozone to Precursor Emission Changes with a Response Surface Model

James T. Kelly [1,*], Carey Jang [1], Shicheng Long [2], Yun Zhu [2], Jia Xing [3,4], Shuxiao Wang [3,4], Benjamin N. Murphy [5] and Havala O.T. Pye [5]

[1] Office of Air Quality Planning and Standards, U.S. Environmental Protection Agency, Research Triangle Park, NC 27711, USA; kelly.james@epa.gov (J.TK.); jang.carey@epa.gov (C.J.)
[2] School of Environment and Energy, South China University of Technology, Guangzhou Higher Education Mega Center, Guangzhou 510006, China; zhuyun@scut.edu.cn (Y.Z.); long.sc@mail.scut.edu.cn (S. L.)
[3] State Key Joint Laboratory of Environmental Simulation and Pollution Control, School of Environment, Tsinghua University, Beijing 100084, China; xingjia@tsinghua.edu.cn (J.X.); shxwang@tsinghua.edu.cn (S.W.)
[4] State Environmental Protection Key Laboratory of Sources and Control of Air Pollution Complex, Beijing 100084, China
[5] Center for Environmental Measurement and Modeling, U.S. Environmental Protection Agency, Research Triangle Park, NC 27711, USA; murphy.ben@epa.gov (B.N.M.); pye.havala@epa.gov (H.O.T.P.)
* Correspondence: kelly.james@epa.gov (J.T.K.)

Text S1. Model Performance Evaluation

CMAQ predictions were evaluated by comparison with observations from monitoring sites. Modeled $PM_{2.5}$ and maximum daily average 8-hr (MDA8) ozone concentrations were compared with available observations from U.S. EPA's Air Quality System (AQS) database (www.epa.gov/aqs). Modeled concentrations of $PM_{2.5}$ components (nitrate; sulfate; elemental carbon; EC; and organic carbon, OC) were compared with observations from the Chemical Speciation Network (CSN) and Interagency Monitoring of Protected Visual Environments (IMPROVE) network [1]. Model predictions were paired with observations in space and time by averaging predictions to the observation sampling period and matching predictions with monitors in a model grid cell. Absolute and normalized bias and error statistics and Pearson correlation coefficients are provided in Table S1.

Performance statistics for this application are generally within the range of model performance statistics reported in previous applications [2,3]. However, $PM_{2.5}$ OC predictions are biased high against observations by >100% in January and contribute to overpredictions of $PM_{2.5}$ concentrations. Running CMAQ with an alternative organic aerosol treatment (i.e., pcSOA rather than nonvolatile POA) did not improve model performance for OC. This behavior suggests that issues with emissions and/or meteorological factors are responsible for the overpredictions, but future work is needed to diagnose the cause of OC biases in January. Overall, the model evaluation suggests that the simulations are suitable for use in our application, but the performance results in Table S1 should be considered in interpreting model results.

Table S1. Model performance statistics (see Table S2 for definition of statistics).

Species	Network	Month	N[a]	Mean Observed[b]	Mean Modeled[b]	MB[a,b]	NMB[a] (%)	RMSE[a,b]	NME[a] (%)	r[a]
$PM_{2.5}$	AQS	January	16940	8.41	11.64	3.23	38.4	6.76	53.5	0.65
		July	16186	8.49	8.68	0.19	2.2	4.63	38.4	0.41
MDA8 O_3	AQS	January	12035	32.70	31.51	-1.19	-3.6	6.01	14.7	0.58
		July	24356	41.83	43.31	1.48	3.6	7.34	13.3	0.80
Sulfate	CSN	January	831	1.17	1.15	-0.02	-1.8	0.75	36.7	0.55
		July	721	1.45	1.08	-0.37	-25.4	0.74	35.1	0.71
	IMPROVE	January	487	0.83	0.78	-0.05	-6.3	0.37	32.6	0.75
		July	441	1.17	0.72	-0.45	-38.3	0.71	44.6	0.66
Nitrate	CSN	January	830	1.91	2.47	0.56	29.6	1.57	53.3	0.75
		July	721	0.31	0.19	-0.12	-38.0	0.36	76.2	0.21
	IMPROVE	January	487	0.90	1.04	0.14	15.5	1.03	70.5	0.60
		July	441	0.18	0.15	-0.04	-20.6	0.18	66.2	0.49
EC	CSN	January	784	0.61	0.75	0.14	22.4	0.67	56.4	0.61
		July	669	0.52	0.42	-0.10	-19.7	0.31	41.6	0.51
	IMPROVE	January	520	0.17	0.27	0.09	52.3	0.25	78.5	0.66
		July	459	0.14	0.14	0.00	0.6	0.22	56.5	0.45
OC	CSN	January	784	1.93	3.92	1.99	102.9	3.42	114.3	0.59
		July	669	1.87	2.95	1.08	57.6	1.91	67.7	0.53
	IMPROVE	January	518	0.81	2.07	1.26	155.1	3.06	160.7	0.45
		July	461	1.33	2.10	0.77	58.1	2.90	88.8	0.12

[a]N: number of samples (site-days); MB: Mean Bias; NMB: Normalized Mean Bias; RMSE: Root Mean Squared Error; NME: Normalized Mean Error; r: Pearson correlation coefficient

[b]$\mu g\ m^{-3}$ for $PM_{2.5}$ and $PM_{2.5}$ components; ppbv for MDA8 O_3

Table S2. Definition of statistics used in the CMAQ model performance evaluation.

Statistic	Description
MB (μg m^{-3}) = $\frac{1}{n}\sum_{i=1}^{n}(P_i - O_i)$	Mean bias (MB) is defined as the average difference between predicted (P) and observed (O) concentrations for the total number of samples (n)
RMSE (μg m^{-3}) = $\sqrt{\sum_{i=1}^{n}(P_i - O_i)^2/n}$	Root mean-squared error (RMSE)
NMB (%) = $\frac{\sum_i^n (P_i - O_i)}{\sum_i^n O_i} \times 100$	The normalized mean bias (NMB) is defined as the sum of the difference between predictions and observations divided by the sum of observed values
NME (%) = $\frac{\sum_i^n \lvert P_i - O_i \rvert}{\sum_i^n O_i} \times 100$	Normalized mean error (NME) is defined as the sum of the absolute value of the difference between predictions and observations divided by the sum of observed values
r = $\frac{\sum_{i=1}^{n}(P_i - \bar{P})(O_i - \bar{O})}{\sqrt{\sum_{i=1}^{n}(P_i - \bar{P})^2}\sqrt{\sum_{i=1}^{n}(O_i - \bar{O})^2}}$	Pearson correlation coefficient

Table S3. Fractional change in U.S. anthropogenic emissions* for 23 simulations used in developing the pf-RSM. A value of 1.000 indicates no change.

Run	NOx	SO_2	NH_3	VOC	$pPM_{2.5}$
1	1.000	1.000	1.000	1.000	1.000
2	0.930	0.838	0.865	0.191	1.000
3	0.043	0.920	0.879	0.372	1.000
4	1.192	1.023	0.037	0.468	1.000
5	0.244	0.657	0.763	0.517	1.000
6	1.175	1.170	1.004	0.894	1.000
7	0.196	0.821	1.148	0.711	1.000
8	0.581	0.163	0.254	0.043	1.000
9	0.175	0.276	0.605	1.041	1.000
10	0.686	0.586	1.095	1.069	1.000
11	0.512	0.023	1.175	0.619	1.000
12	0.433	0.041	0.189	0.979	1.000
13	1.094	0.635	0.427	0.636	1.000
14	0.778	0.447	0.084	0.768	1.000
15	0.714	0.552	0.633	1.195	1.000
16	0.794	0.312	0.366	0.163	1.000
17	0.420	0.259	0.978	0.174	1.000
18	0.226	0.088	0.522	0.061	1.000
19	1.052	0.978	0.649	0.971	1.000
20	0.865	0.792	0.131	0.259	1.000
21	0.000	0.000	0.000	0.000	1.000
22	1.000	1.000	1.000	1.000	0.000
23	1.000	1.000	1.000	1.000	0.500

*Modeled U.S. anthropogenic emission sectors: PT_EGU, PT_NONEGU, PT_OILGAS, PT_CMV_C3, PT_CMV_C1C2, GRIDDED_AFDUST_ADJ, GRIDDED_AG, GRIDDED_NONPT, GRIDDED_NONROAD, GRIDDED_NP_OILGAS, GRIDDED_ONROAD, GRIDDED_PT_OILGAS, GRIDDED_PTNONIPM, GRIDDED_RAIL, and GRIDDED_RWC

Table S4. Fractional change in U.S. anthropogenic emissions for 30 OOS simulations used in evaluating the pf-RSM.

Run	NOx	SO_2	NH_3	VOC
1	0.975	0.960	1.164	0.018
2	1.039	0.753	0.119	0.494
3	0.914	0.358	0.550	0.473
4	0.403	0.347	0.021	0.190
5	0.027	0.102	0.761	0.189
6	0.802	0.172	0.680	0.554
7	0.364	1.182	0.480	0.725
8	0.538	0.992	0.341	0.861
9	0.699	0.244	0.777	0.421
10	0.290	0.695	0.487	1.059
11	1.000	1.000	0.800	1.000
12	1.000	1.000	0.600	1.000
13	1.000	1.000	0.400	1.000
14	1.000	1.000	0.200	1.000
15	1.000	1.000	0.000	1.000
16	0.800	1.000	1.000	1.000
17	0.600	1.000	1.000	1.000
18	0.400	1.000	1.000	1.000
19	0.200	1.000	1.000	1.000
20	0.000	1.000	1.000	1.000
21	1.000	0.800	1.000	1.000
22	1.000	0.600	1.000	1.000
23	1.000	0.400	1.000	1.000
24	1.000	0.200	1.000	1.000
25	1.000	0.000	1.000	1.000
26	1.000	1.000	1.000	0.800
27	1.000	1.000	1.000	0.600
28	1.000	1.000	1.000	0.400
29	1.000	1.000	1.000	0.200
30	1.000	1.000	1.000	0.000

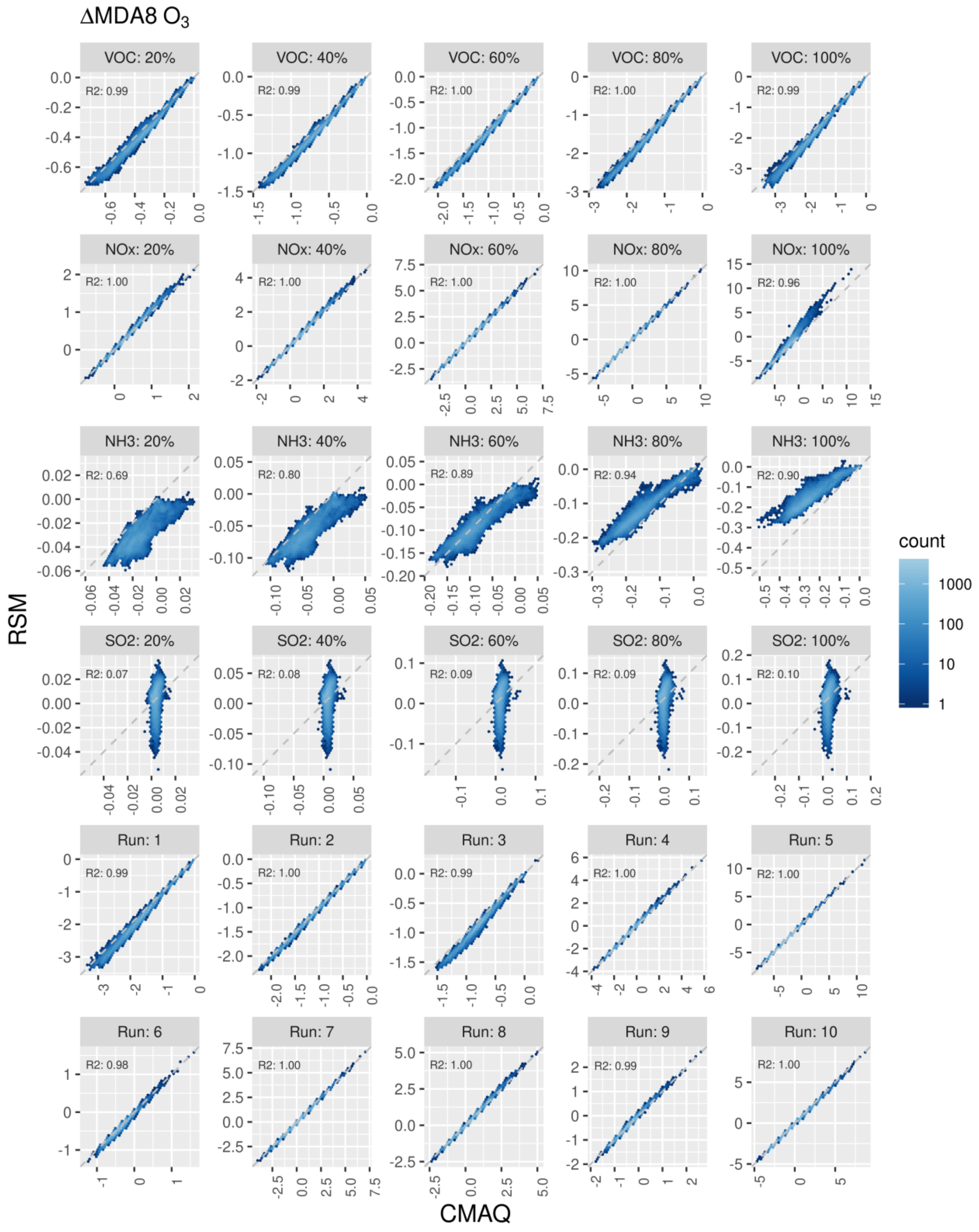

Figure S1. Comparison of changes in mean January MDA8 ozone concentrations predicted by the pf-RSM and 30 OOS CMAQ simulations. Units: ppb.

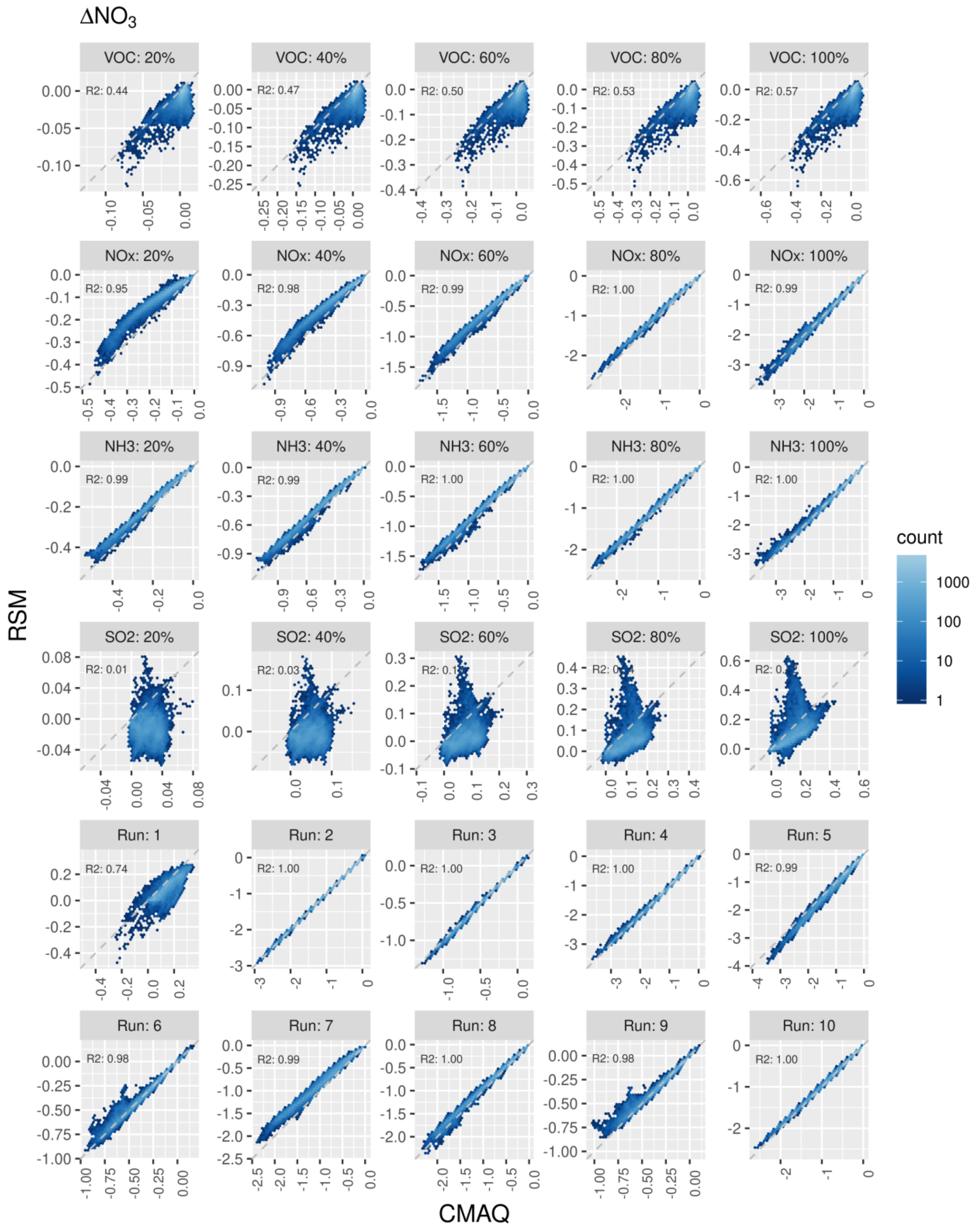

Figure S2. Comparison of changes in mean January nitrate concentrations predicted by the pf-RSM and 30 OOS CMAQ simulations. Units: μg m^{-3}.

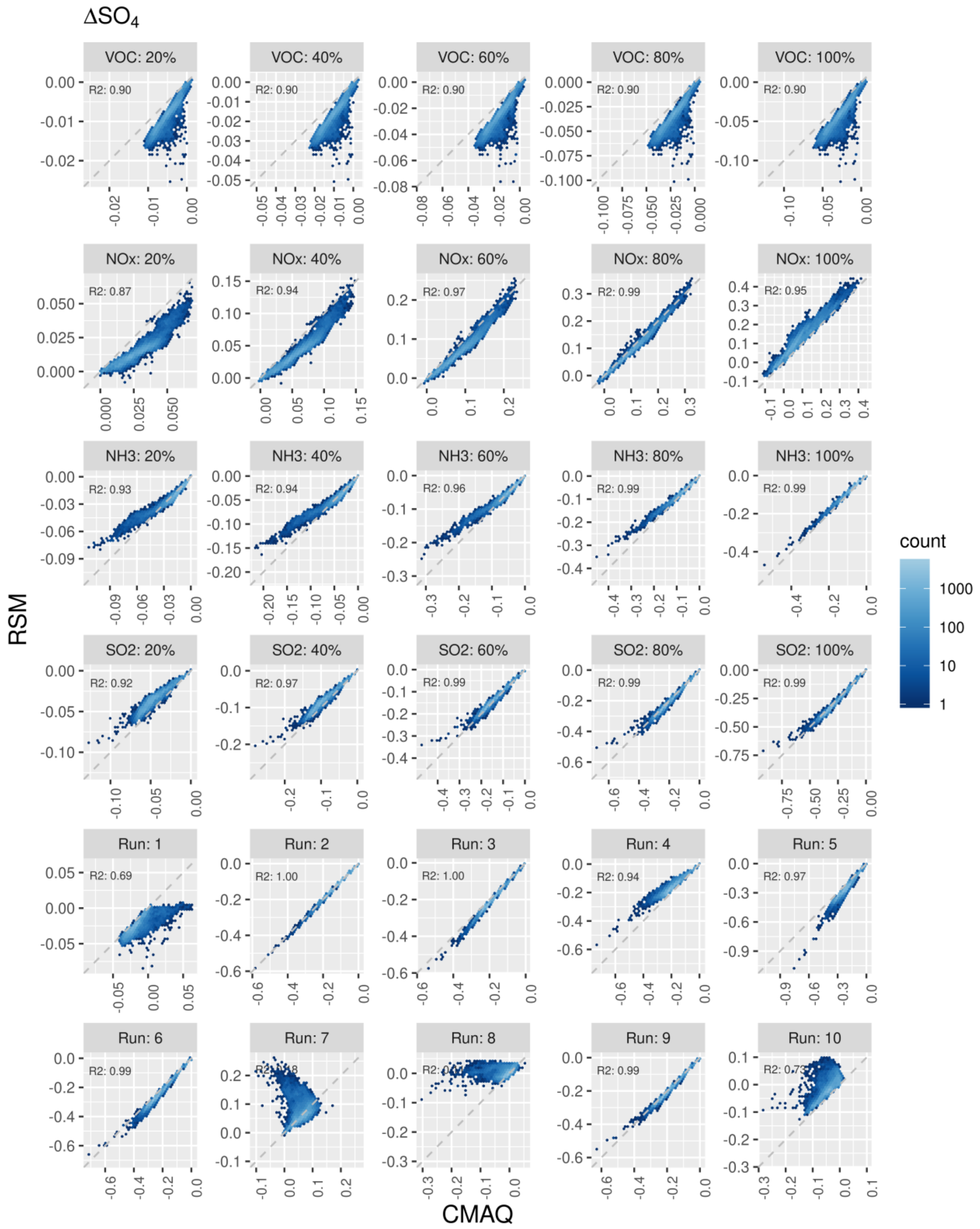

Figure S3. Comparison of changes in mean January sulfate concentrations predicted by the pf-RSM and 30 OOS CMAQ simulations. Units: μg m-3.

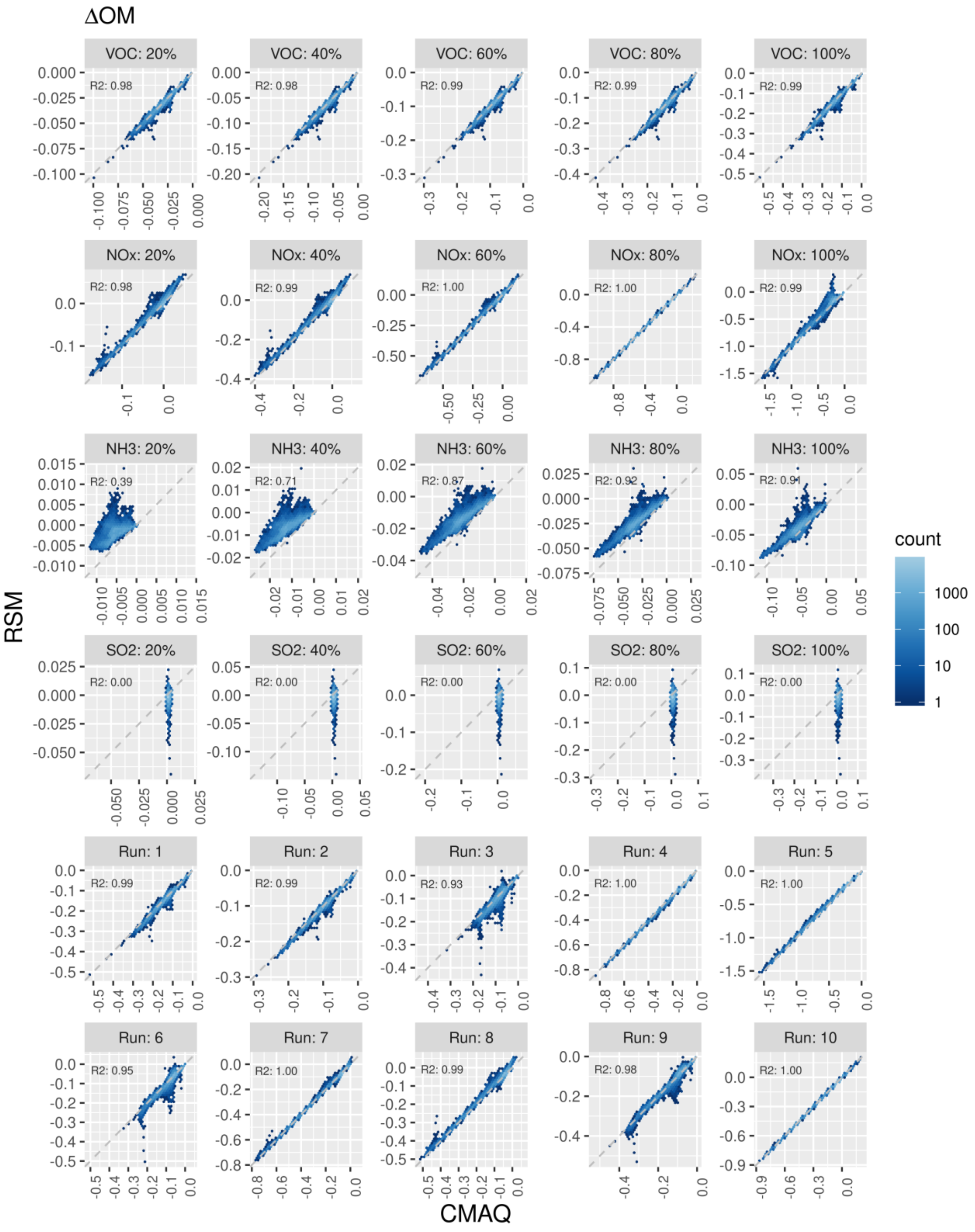

Figure S4. Comparison of changes in mean January OM concentrations predicted by the pf-RSM and 30 OOS CMAQ simulations. Units: μg m^{-3}.

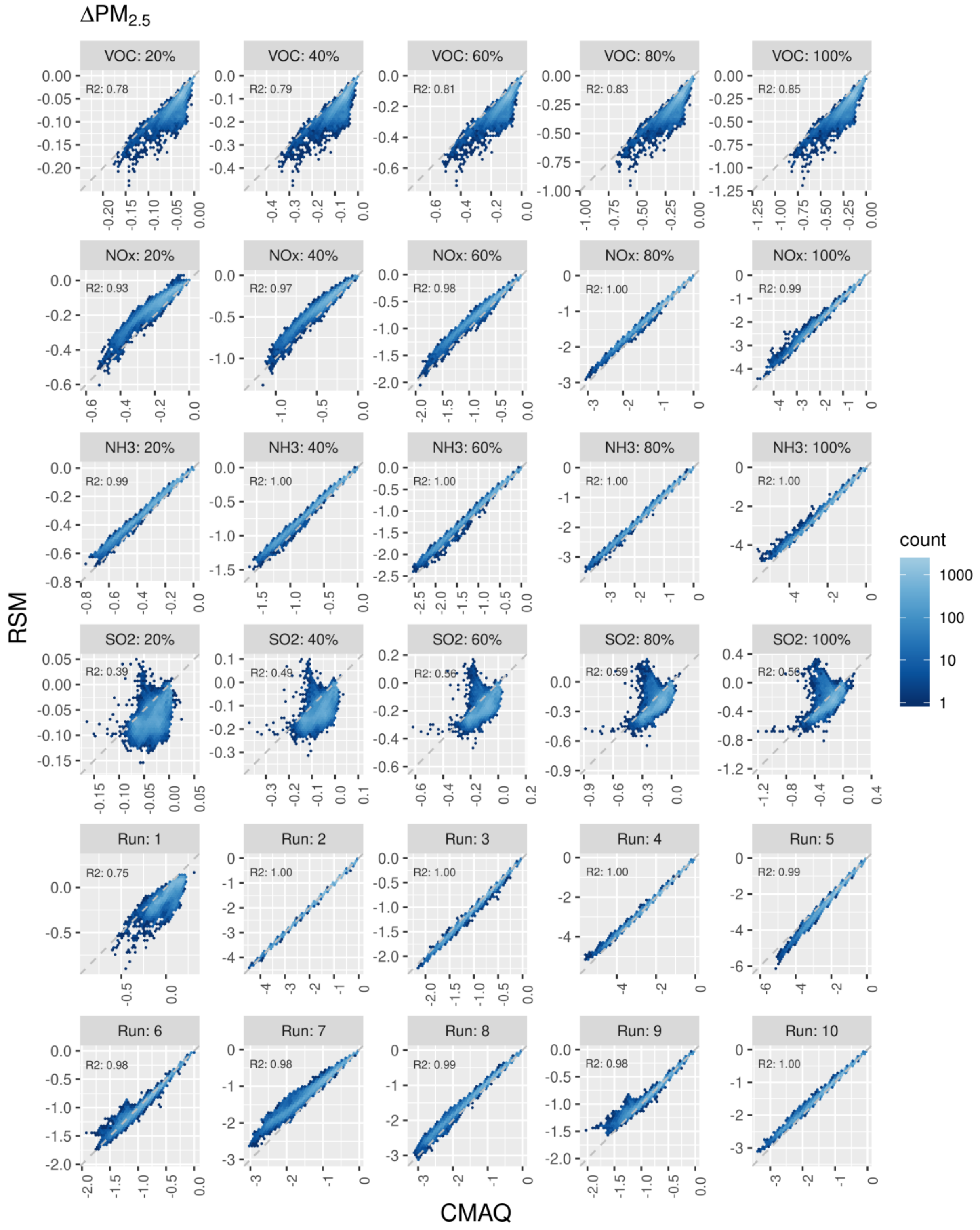

Figure S5. Comparison of changes in mean January $PM_{2.5}$ concentrations predicted by the pf-RSM and 30 OOS CMAQ simulations. Units: µg m^{-3}.

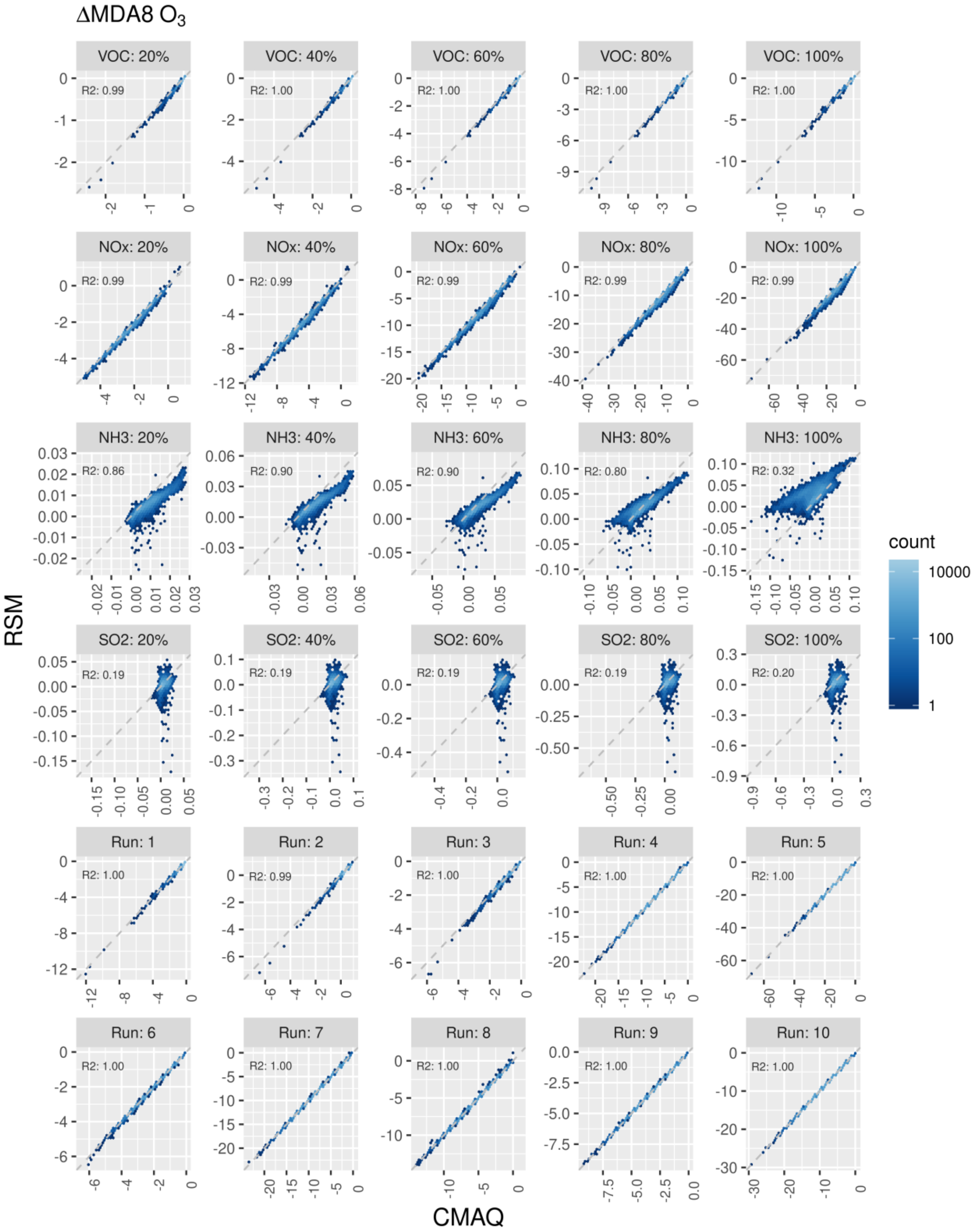

Figure S6. Comparison of changes in mean July MDA8 ozone concentrations predicted by the pf-RSM and 30 OOS CMAQ simulations. Units: ppb.

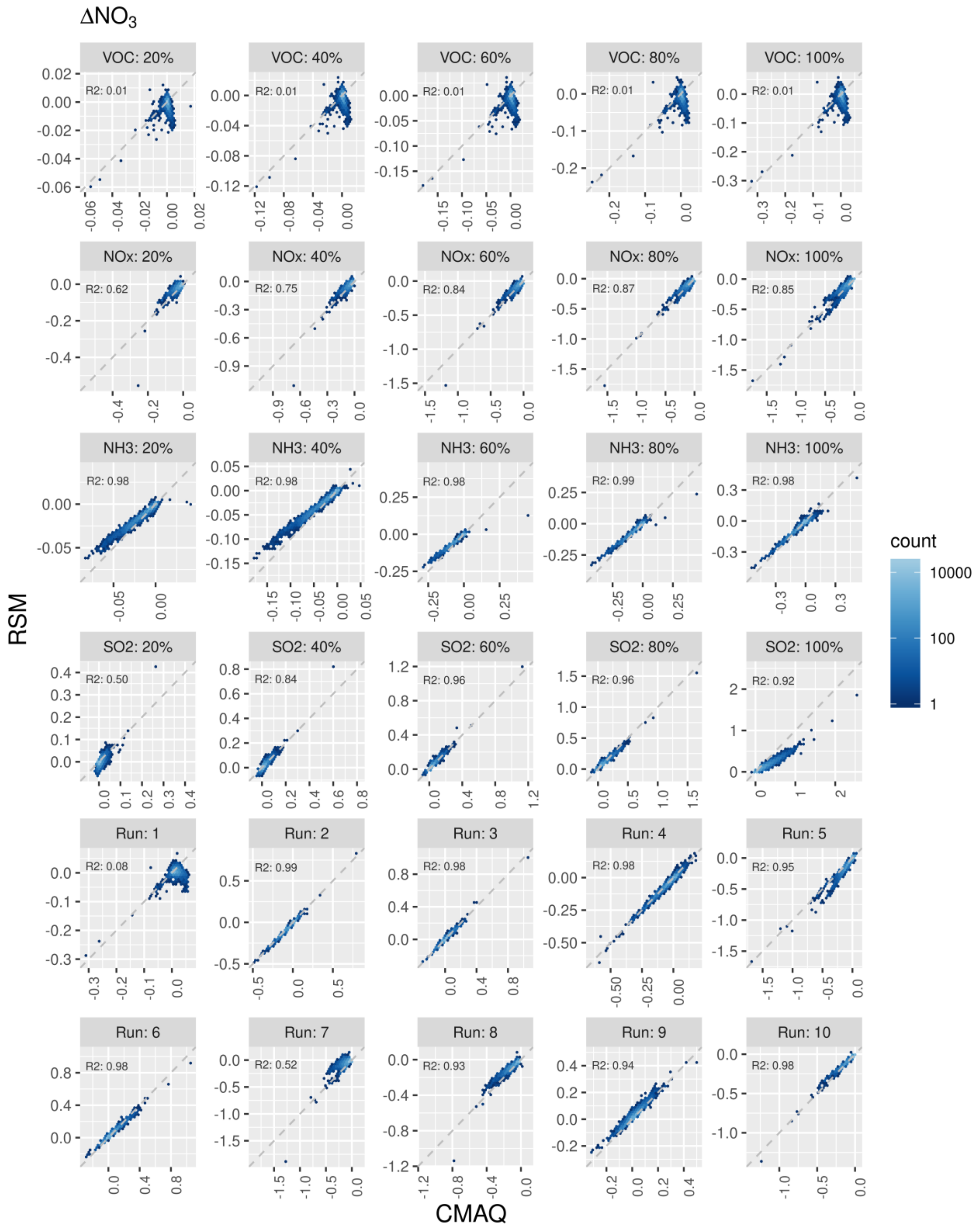

Figure S7. Comparison of changes in mean July nitrate concentrations predicted by the pf-RSM and 30 OOS CMAQ simulations. Units: µg m^{-3}.

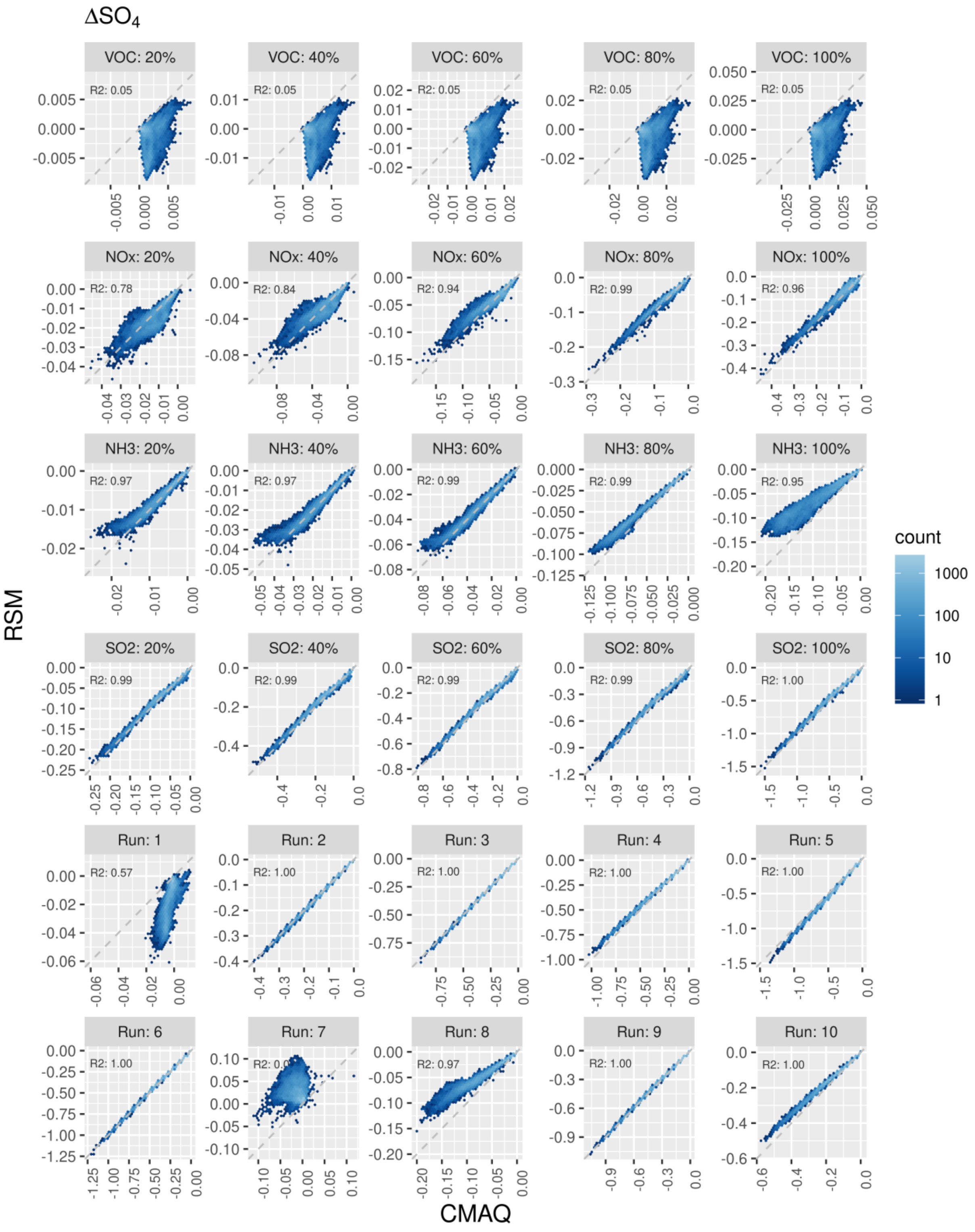

Figure S8. Comparison of changes in mean July sulfate concentrations predicted by the pf-RSM and 30 OOS CMAQ simulations. Units: µg m^{-3}.

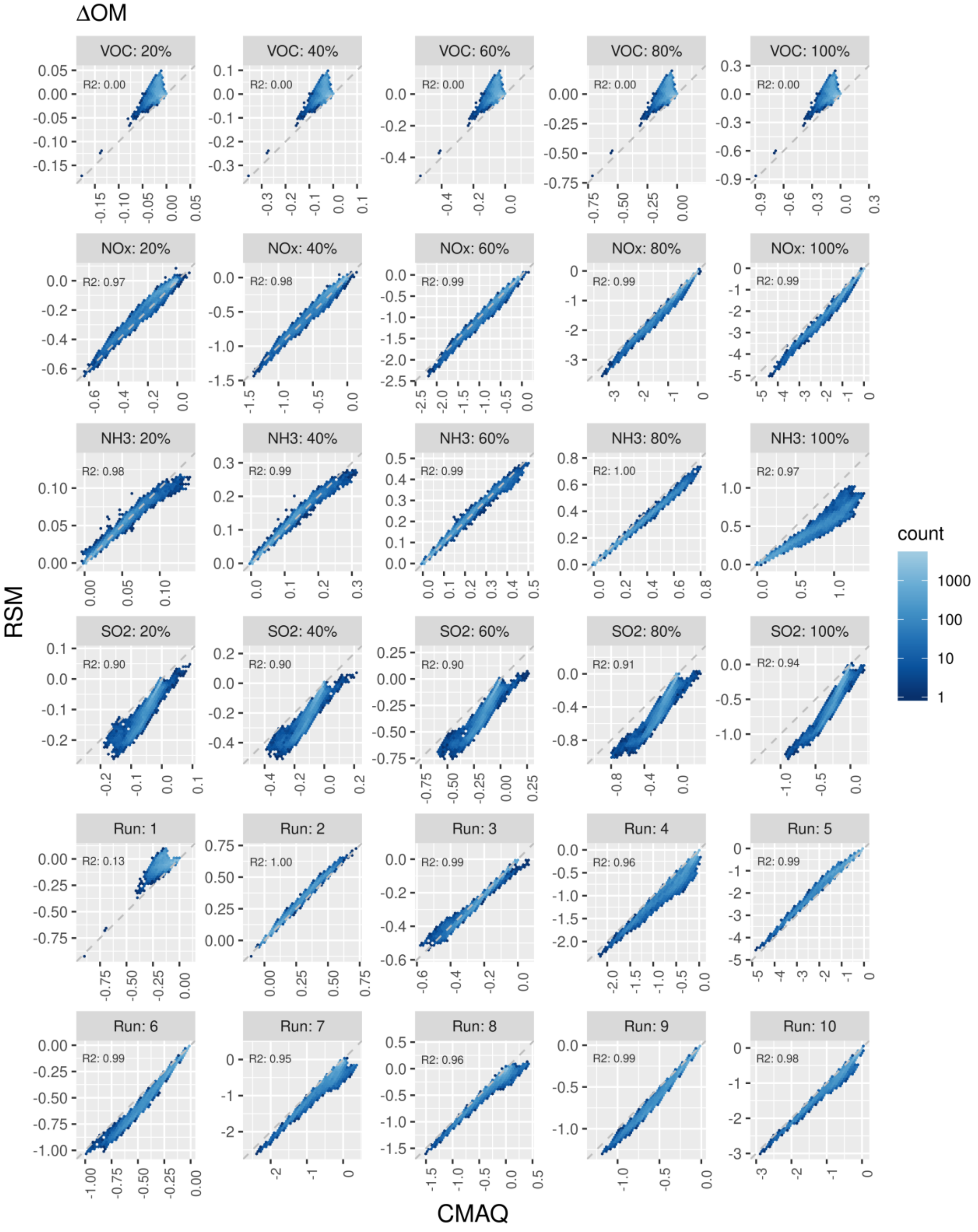

Figure S9. Comparison of changes in mean July OM concentrations predicted by the pf-RSM and 30 OOS CMAQ simulations. Units: μg m^{-3}.

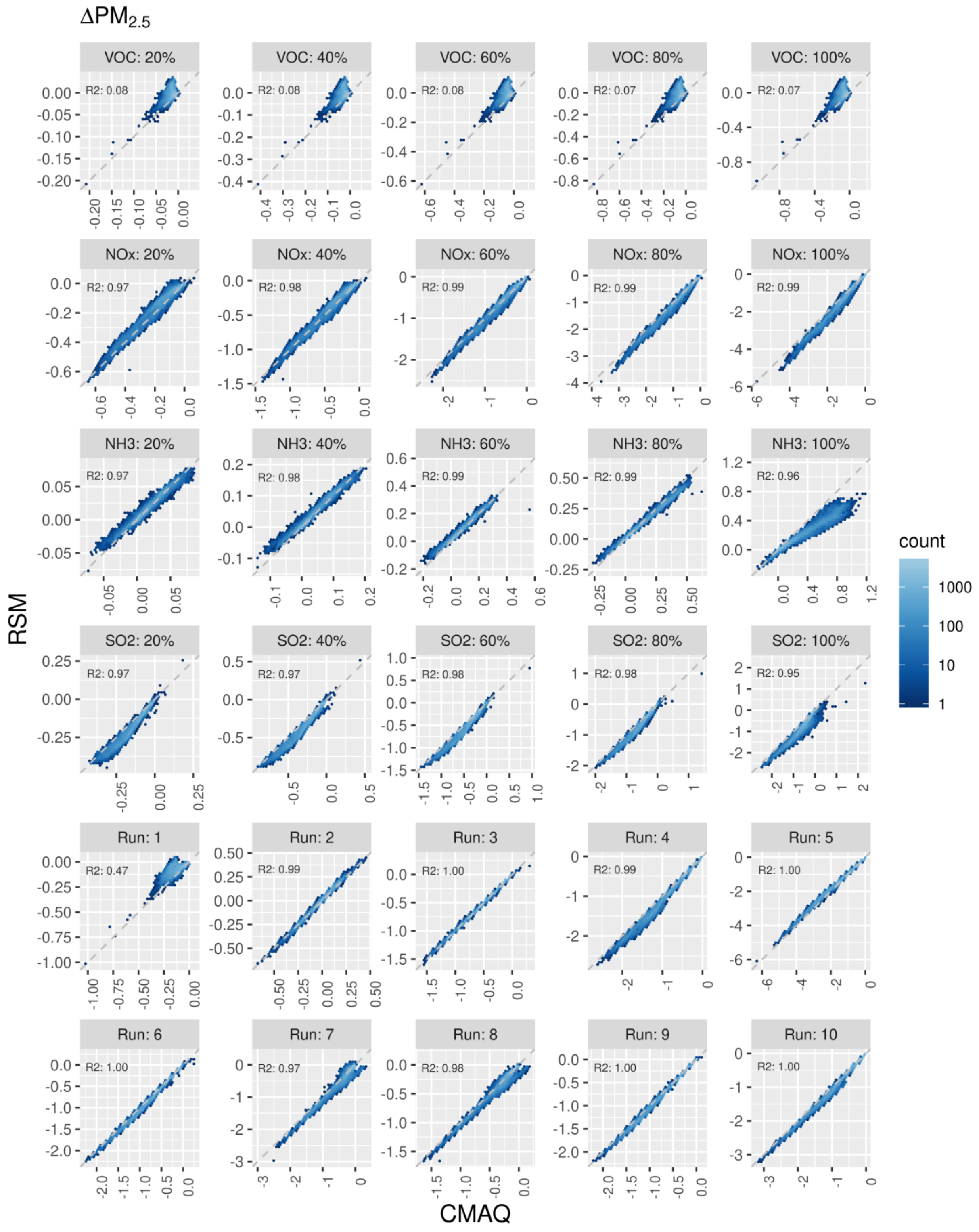

Figure S10. Comparison of changes in mean July $PM_{2.5}$ concentrations predicted by the pf-RSM and 30 OOS CMAQ simulations. Units: μg m^{-3}.

References

1. Solomon, P.A.; Crumpler, D.; Flanagan, J.B.; Jayanty, R.K.M.; Rickman, E.E.; McDade, C.E. US National PM2.5 Chemical Speciation Monitoring Networks-CSN and IMPROVE: Description of networks. *Journal of the Air & Waste Management Association* **2014**, *64*, 1410-1438, doi:10.1080/10962247.2014.956904.
2. Kelly, J.T.; Koplitz, S.N.; Baker, K.R.; Holder, A.L.; Pye, H.O.T.; Murphy, B.N.; Bash, J.O.; Henderson, B.H.; Possiel, N.C.; Simon, H., et al. Assessing PM2.5 model performance for the conterminous U.S. with comparison to model performance statistics from 2007-2015. *Atmospheric Environment* **2019**, *214*, 116872, doi:10.1016/j.atmosenv.2019.116872.
3. Simon, H.; Baker, K.R.; Phillips, S. Compilation and interpretation of photochemical model performance statistics published between 2006 and 2012. *Atmospheric Environment* **2012**, *61*, 124-139, doi:10.1016/j.atmosenv.2012.07.012.
